# Comprehensive comparative analysis of histopathology and gene expression in subchondral bone between kashin-beck disease and primary osteoarthritis

**DOI:** 10.3389/fgene.2022.942326

**Published:** 2022-07-18

**Authors:** Lei Yang, Jingwen Sun, Ying Zhang, Xiong Guo, Guanghui Zhao

**Affiliations:** ^1^ School of Nursing, Health Science Center, Xi’an Jiaotong University, Xi’an, China; ^2^ School of Public Health, Health Science Center, Key Laboratory of Trace Elements and Endemic Diseases of National Health and Family Planning Commission, Collaborative Innovation Center of Endemic Diseases and Health Promotion in Silk Road Region, Xi’an Jiaotong University, Xi’an, China; ^3^ Department of Joint Surgery, Hong Hui Hospital, Xi’an Jiaotong University Health Science Center, Xi’an, China

**Keywords:** kashin-beck disease, osteoarthritis, subchondral bone, histopathology, RNA sequencing, microRNA array, differentially expressed genes

## Abstract

Kashin-Beck disease (KBD) is an endemic, degenerative osteoarthropathy that exhibits some similar characteristics to osteoarthritis (OA) but with different etiologies and pathogeneses. In addition to cartilage damage, microstructural changes of bone were observed in KBD. This study aimed to comparatively demonstrate the general histopathological changes, transcriptomics, and differentially expressed miRNAs of subchondral bone between KBD and OA. Tibial plateau subchondral bone samples were collected from eighteen patients with KBD and eighteen patients with OA. Histopathological changes were examined by hematoxylin-eosin (HE) staining, safranin O-fast green staining, and picrosirius red staining. RNA sequencing and miRNA array analysis were performed to screen the differentially expressed genes (DEGs) and differentially expressed miRNAs (DEMs), respectively. The subchondral bone samples of the tibial plateau of KBD and OA both showed increased thickness and sclerosis. A total of 179 DEGs and 124 DEMs were identified in subchondral bone between KBD and OA, which were involved in several vital GO terms and KEGG signaling pathways. Our results suggest that the pathological mechanisms of subchondral bone are different between KBD and OA, although they exhibit similar histopathological features. Integrated analysis revealed several genes such as ADAMTS14, SLC13A5, and CEACAM1, that may be crucial DEGs in subchondral bone between KBD and OA, suggesting that these genes could serve as potential differential diagnostic biomarkers for subchondral bone lesions in KBD and OA. These findings provide valuable information for further clarifying pathological changes in subchondral bone in KBD and OA.

## Introduction

Kashin-Beck disease (KBD) is an endemic, chronic osteoarthropathy that has a high prevalence in regions of eastern Siberia, northeast to southwest China and North Korea (Guo et al., 2014). Although the incidence of KBD has decreased significantly in recent years, there are still more than 0.18 million individuals suffering from KBD in China according to the China Health Statistical Yearbook 2020 (National Health Commission of the People’s Republic of China, 2020). The etiology of KBD is still unclear, and many studies have shown that KBD is a complex disorder that is caused by the interaction of multiple environmental risk factors (such as selenium deficiency and mycotoxin contamination) and genetic components (Yang, 1995; Lu et al., 2012; Yang et al., 2016). KBD causes lesions mainly in the articular and epiphyseal plate cartilage tissues of multiple joints that lead to different degrees of joint deformation and restriction of movement of affected individuals (Mathieu et al., 1997).

Osteoarthritis (OA) is the most common age-related degenerative joint disorder and is a major cause of pain and disability in the population worldwide (Glyn-Jones et al., 2015). OA is characterized by cartilage degradation, bone remodeling, and osteophyte formation that leads to clinical manifestations, including pain, stiffness, swelling, and limitations in joint function (Allen et al., 2021). Although adult KBD patients present some similar characteristics to OA patients, they are distinctly different in terms of age of onset, etiology, and pathogenesis. In contrast to OA, the symptoms of KBD patients, such as joint deformation, usually start to appear in children at the age of 5–15 years, and the joints of most adult KBD patients become more severely damaged as the disease progresses (Moreno-Reyes et al., 2001). Recent comparative studies have demonstrated several differentially expressed genes (Duan et al., 2010) and differentially methylated regions (Fan et al., 2021) in the cartilage between KBD and OA, confirming that the pathological mechanisms of the two diseases were different.

Subchondral bone is a bone area located below the articular cartilage and composed of the subchondral bone plate and the subchondral trabecular bone (Goldring and Goldring, 2010). Although the hallmark of OA remains a progressive degradation of cartilage, increasing evidence shows that subchondral bone plays a crucial role in the initiation and progression of OA (Lambova and Müller-Ladner, 2018). Subchondral bone is considered to provide support for the overlying articular cartilage, and its lesions are commonly associated with articular cartilage defects (Madry et al., 2010). Subchondral bone is a transforming zone that provides a direct link between cartilage and deeper bone, which is important for cartilage nutrient supply and metabolism. Pathologically, the onset of KBD is characterized by chondrocyte necrosis in deep zones of cartilage (Guo et al., 2014); a potential mechanism is that subchondral bone alterations may contribute to this distinctive pathological characteristic in KBD. However, current studies on KBD subchondral bone are lacking.

Strong evidence has revealed many microarchitectural and histopathological changes in the subchondral bone of the OA joint that lead to OA progression (Li G. et al., 2013). A recent radiographic study also showed that the bone texture structure in KBD had clearly changed in subchondral and trabecular bone, but interestingly, these microstructural changes were not identical to OA (Li et al., 2018). In the present study, we first clarified the general histopathological characteristics of subchondral bone in the tibial plateau of KBD and compared its similarities and differences with OA. Then, we compared the transcriptomics and differentially expressed microRNAs (miRNAs) of subchondral bone between KBD and OA and further explored the key differentially expressed genes between them by integrated analysis. Taken together, we focused on subchondral bone in KBD and OA, to provide new findings on the pathological mechanism of KBD, and to explore the potential genetic differential diagnostic biomarkers between KBD and OA.

## Materials and methods

### Sample collection

**FIGURE 1:**
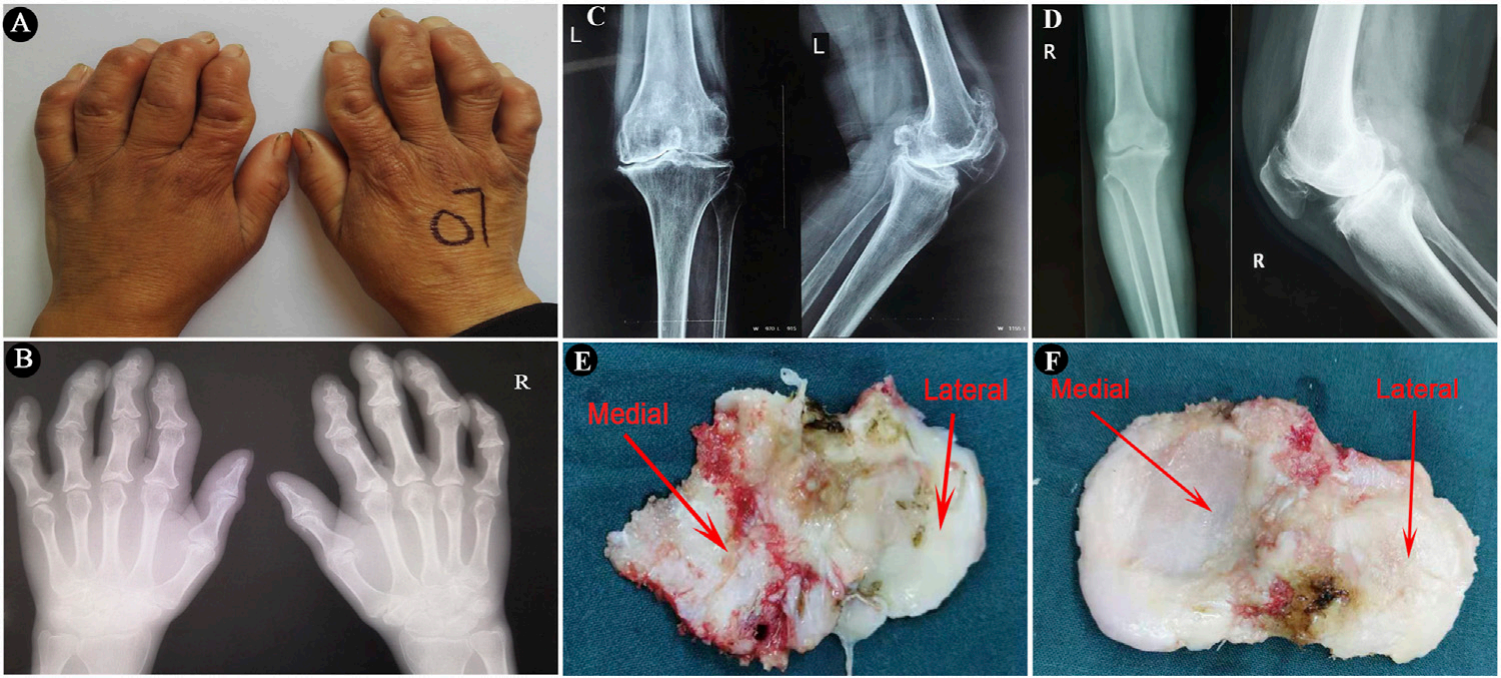
The imaging characteristics of the included subjects and surgically collected specimens. **(A,B)**, Images and radiographic images of bilateral hands of a KBD patient, showing crooked fingers and metaphyseal lesions in the phalanges. **(C,D)**, Radiographic images of anteroposterior and lateral axial X-ray examination of knee joints of a KBD patient and an OA patient, respectively. **(E,F)**, Images of surgically collected specimens of tibial plateaus from a KBD patient and an OA patient, respectively.

The study protocol was approved by the Ethics Committee of the Health Science Center, Xi’an Jiaotong University. The diagnosis of KBD was according to the diagnostic criteria of WS/T 207–2010 (National Health Commission of the People’s Republic of China, http://www.nhc.gov.cn/wjw/s9500/201006/47920.shtml). The diagnosis of OA was based on the American College of Rheumatology criteria (Altman et al., 1986). Tibial plateaus were collected from eighteen patients with KBD (mean age 61.4 ± 4.8 years) and eighteen patients with OA (mean age 65.3 ± 4.1 years) when they were treated with total knee arthroplasty at Xi’an Honghui Hospital, China. Five paired samples from KBD and OA were used for the histological assay, while nine paired samples were used for mRNA sequencing and four paired samples were used for miRNA array profiling analysis. All patients accepted anteroposterior and lateral axial X-ray examination of knee joints before the surgery. The clinical and radiographic characteristics of the hand and knee joints of the included subjects are shown in Figure 1. Classification of knee severity of patients with KBD and OA was performed using the Kellgren-Lawrence (KL) grading system. The basic characteristics of the recruited participants are shown in Supplementary Table S1.

### Histological processing

The tibial plateau samples were first separated into medial tibial (MT) and lateral tibial (LT) parts by an electrosurgical knife after surgical collection (Figure 1). All of the specimens for histological and immunohistochemical analyses were washed in saline and fixed in 4% paraformaldehyde immediately for 48 h, followed by decalcification in 10% ethylenediaminetetraacetic acid (EDTA) solution for 4 weeks. The decalcified specimens were dehydrated in ascending grades of alcohol and embedded in paraffin with a consistent spatial orientation. The embedded specimens were then sectioned into 5-μm-thick slices along the sagittal plane. The histopathological changes in the osteochondral tissues from KBD and OA tibial plateaus were observed by hematoxylin-eosin (HE) staining, safranin O-fast green staining, and picrosirius red staining according to the recommended protocols (Schmitz et al., 2010). The sections were scanned with a P250 scanner (3DHistech, Hungary) and imaged using CaseViewer 2.0 (3DHistech). The picrosirius red-stained sections were viewed and photographed under a polarized light microscope (OLYMPUS, Japan).

### Sample preparation and total RNA extraction

For mRNA sequencing and miRNA array analysis, all specimens were prepared from the medial plateaus to ensure consistency of sampling regions between the KBD and OA groups. Following the collection of the medial tibial plateaus, the articular cartilage and trabecular bone were carefully eliminated using a scalpel under a microscope. Then, the subchondral bone tissues were cut into pieces and immediately flash-frozen in liquid nitrogen to prevent RNA degradation. The frozen specimens were disrupted using a mortar and pestle in liquid nitrogen for RNA extraction. Total RNA was extracted using the RNeasy Mini Kit according to the manufacturer’s instructions (Qiagen, Germany). Immediately following the extraction, the quality and quantity of total RNA were determined with a Nano Drop™ One device (Thermo Scientific, United States). The integrity of total RNA was assessed by visualization of the 28S/18S banding pattern using agarose gel electrophoresis.

### RNA sequencing and data analysis

Illumina platform sequencing of all samples was conducted following a standard protocol at CapitalBio Technology Co., Ltd., Beijing. In brief, mRNA was purified from total RNA using Oligo (dT) magnetic beads. Fragmentation was performed in NEBNext First Strand Synthesis Reaction Buffer (5X). First-strand cDNA was synthesized using ProtoScript II Reverse Transcriptase. This was followed by second-strand cDNA synthesis using Second Strand Synthesis Reaction Buffer (×10) and Second Strand Synthesis Enzyme Mix. cDNA fragments were subjected to end repair and ligation of sequencing adapters, followed by PCR amplification to create the final cDNA library. Finally, sequencing was performed using the Illumina NovaSeq 6000 platform (Illumina, San Diego, CA, United States).

Raw sequencing reads in FASTQ format were first processed using fastp software (Chen et al., 2018), and clean reads were obtained by removing reads containing adapter, ploy-N, or reads with low-quality, followed by quality checking using FastQC software (Andrews, 2010). These clean reads were then mapped to the reference genome using HISAT2 software (Kim et al., 2015). StringTie was used for transcript reconstruction (Pertea et al., 2015). FeatureCounts and StringTie were used to quantify the read count and expression abundance of fragments per kilobase per million reads (FPKM) of each transcript (Liao et al., 2014; Pertea et al., 2015). Taxonomic and functional annotations of the expressed genes were obtained by comparing with five databases including, ENSEMBL, NCBI, Uniprot, Kyoto Encyclopedia of Genes and Genomes (KEGG) and Gene Ontology (GO).

### Differential expression and enrichment analyses

The differentially expressed genes (DEGs) between the two groups were identified with |log2-fold change| ≥1 and P value ≤0.05 using the limma package (Ritchie et al., 2015). Hierarchical cluster analysis was performed on the screened DEGs by the R package. GO function and KEGG pathway enrichment analyses of the DEGs were performed using KOBAS 2.0 (Xie et al., 2011). A hypergeometric test was applied to identify a GO term or KEGG pathway that was significantly enriched in DEGs. Finally, the protein-protein interactions (PPIs) of the DEGs were constructed using the STRING database and visualized by Cytoscape software (Szklarczyk et al., 2015).

### MicroRNA array profiling and data analysis

Affymetrix miRNA 4.0 Array was used to perform miRNA array profiling as recommended by the manufacturer at CapitalBio Technology Co., Ltd., Beijing. Briefly, the total RNA was first labeled with biotin using a Genisphere FlashTag labeling kit (Genisphere Inc., Hatfield, PA, United States). Each labeled sample was then hybridized with Affymetrix miRNA 4.0 arrays in a Hybridization Oven 640. After hybridization, the slides were washed and stained on a Fluidics Station 450 and then scanned with a GeneChip® Scanner 3000 (all from Affymetrix, Inc.).

Statistical analysis of miRNA data was performed using Affymetrix^®^ GeneChip^®^ Command Console^®^ (AGCC) software. Expression data were preprocessed, including summarization, background correction, and normalization, using a robust multiarray average algorithm (Irizarry et al., 2003). The differentially expressed miRNAs (DEMs) were identified with a fold change ≥2 or ≤0.5 and q-value <0.05 with a significance analysis of the microarray (SAM) method by the R package. The screened miRNAs were then predicted for target genes using 12 different prediction programs, including miRWalk, Microt4, miRanda, mirbridge, miRDB, miRMap, miRNAMap, Pictar2, PITA, RNA22, RNAhybrid, and TargetScan. Prediction targets were selected only if at least six programs were able to predict simultaneously.

### Quantitative real-time PCR validation of miRNAs

Six miRNAs were selected to validate the RNA-sequencing data. Total RNA was reverse-transcribed into cDNA with the miRNA-specific stem-loop Megaplex RNA reverse transcription primer mixture. qRT-PCR amplification for miRNAs was performed using a QuantStudioTM 7 Flex RealTime PCR system (Applied Biosystems, United States). The 20 µl reaction mixture contained 10 µl 2×miRcute Plus miRNA Premix, 0.4 µl universal reverse primer (200 nM), 0.4 µl miRNA sequence-specific forward primer (200 nM), 2 µl cDNA, and 7.2 µl nuclease-free water. The qRT-PCR was programmed at 95 °C for 10 min, followed by 40 cycles of 95°C for 15 s and 60°C for 1 min. The miRNA primers information is shown Supplementary Table S2. The expression level was determined using the 2^−∆∆CT^ method (Livak and Schmittgen, 2001) and miR-16 was used as an endogenous normalization control. The miR-16 has been used to quantify miRNAs in serum from healthy subjects and large B-cell lymphoma patients (Lawrie et al., 2008), in plasma from tongue squamous cell carcinoma patients (Wong et al., 2008), and in serum from healthy controls and patients with osteogenesis imperfect (Wang et al., 2012). Moreover, the miR-16 is stably expressed between lymphoblastoid cell lines, primary bone marrow aspirates and archived bone marrow samples (Morenos et al., 2012). In addition, it was found that miR16 is a stable and suitable endogenous reference gene for miRNA studies of urinary exosomes derived from patients with chronic kidney disease (Lange et al., 2017).

### Integrated analysis of transcriptomics and miRNA profiling data

Integration of the DEGs from RNA sequencing and the DEMs from microarray was performed to screen candidate DEGs and their miRNAs. If a miRNA was negatively correlated with its target mRNA (a DEG in RNA sequencing), the mRNA was identified as a candidate target DEG of the miRNA. To identify all possible miRNA-mRNA interactions, the obtained miRNAs and target genes were then visualized by Cytoscape 3.8.0 software to construct miRNA-mRNA regulatory networks.

### Immunohistochemistry

Immunohistochemical staining was used to verify the expression of two selected genes, *ADAMTS14* and *SLC13A5*, in subchondral bone from patients with KBD and OA. The primary rabbit anti- ADAMTS14 antibody (ab198885) was purchased from Abcam (Cambridge, United Kingdom), and the primary rabbit anti-SLC13A5 antibody (bs-19796R) was purchased from Beijing Biosynthesis Biotechnology Co., Ltd. (Beijing, China). The rabbit SP detection kit (biotin-streptavidin HRP detection systems, SP-9001) was purchased from ZSGB-BIO (Beijing, China). Briefly, the paraffin-embedded samples were cut into 5-μm-thick paraffin sections and fixed on glass slides for immunohistochemical staining. Then, the paraffin sections were deparaffinized in xylene and rehydrated in gradient alcohol. Endogenous peroxidase activity was blocked with 3% hydrogen peroxide for 10 min at 37°C, and antigen retrieval was performed with a complex digestion solution (AR0022, Boster, Wuhan, China) for 20 min at 37°C. After blocking with 5% (v/v) goat serum for 20 min at room temperature, the sections were incubated with antibody solution and PBS as a negative control overnight at 4°C. After washing, the sections were incubated with biotin-labeled secondary antibody and horseradish peroxidase-labeled streptavidin for 20 min at 37°C. Thereafter, the sections were stained with 3,3′-diaminobenzidine (DAB), followed by hematoxylin counterstaining. Finally, the sections were dehydrated and mounted. The sections were scanned with a P250 scanner (3DHistech, Hungary) and photographed using CaseViewer 2.0 (3DHistech).

## Results

### Histological examination results

**FIGURE 2:**
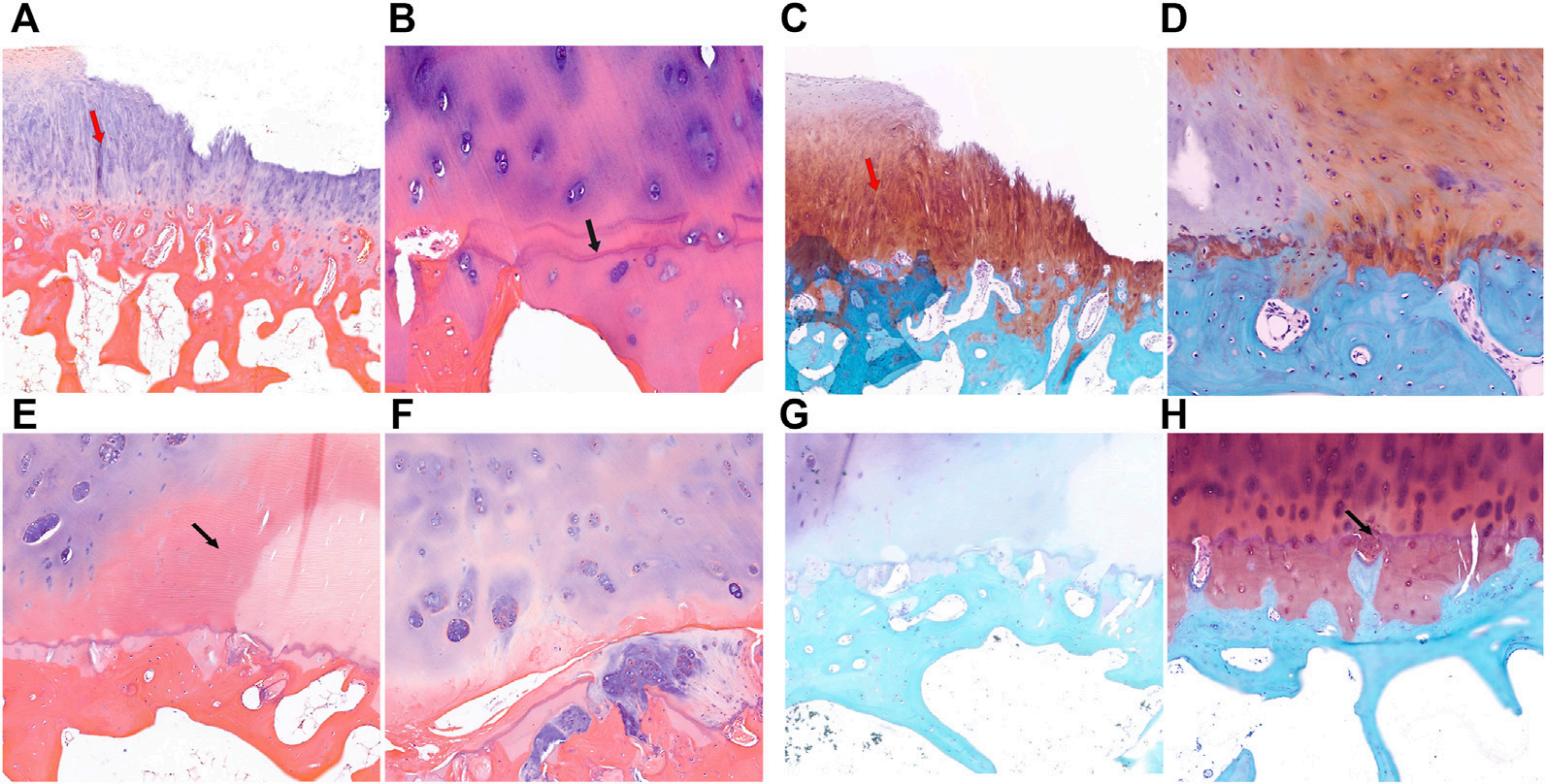
HE and safranin O-fast green staining of cartilage and subchondral bone of the tibial plateau in KBD patients. **(A,B)**, HE staining of the medial tibial plateau. The red arrow shows cartilage fibrosis, and the black arrow shows tidemark replication (magnification: **(A)**, ×5; **(B)**, ×10). C-D, Safranin O-fast green staining of the medial tibial plateau (magnification: **(C)**, ×5; **(D)**, ×10). **(E,F)**, HE staining of the lateral tibial plateau. The black arrow shows chondrocyte necrosis and ossification (magnification: **(E)**, ×5; **(F)**, ×10). **(G,H)**, Safranin O-fast green staining of the lateral tibial plateau. The black arrow shows vascular invasion (magnification: **(G)**, ×5; **(H)**, ×10).

HE staining and safranin O-fast green staining showed significant cartilage damage of the tibial plateau in KBD patients, with evident cartilage defects, thinning or loss, reduction of chondrocytes, and cartilage fibrosis. There were typical chondrocyte necrosis foci in the deep layer of KBD articular cartilage. The tidemark at the base of articular cartilage was displaced anteriorly and duplicated, incomplete, or even absent. There was vascular invasion of the layer of calcified cartilage, and the thickness of the calcified layer was reduced or even absent. The subchondral bone plate and trabeculae were significantly thickened, with pannus-like tissue formation and invasion into the cartilage layer. Overall, the articular cartilage damage and subchondral bone modifications in the medial tibial plateau (Figures 2A–D) were more severe than those on the lateral side (Figures 2E–H) in KBD patients.

**FIGURE 3:**
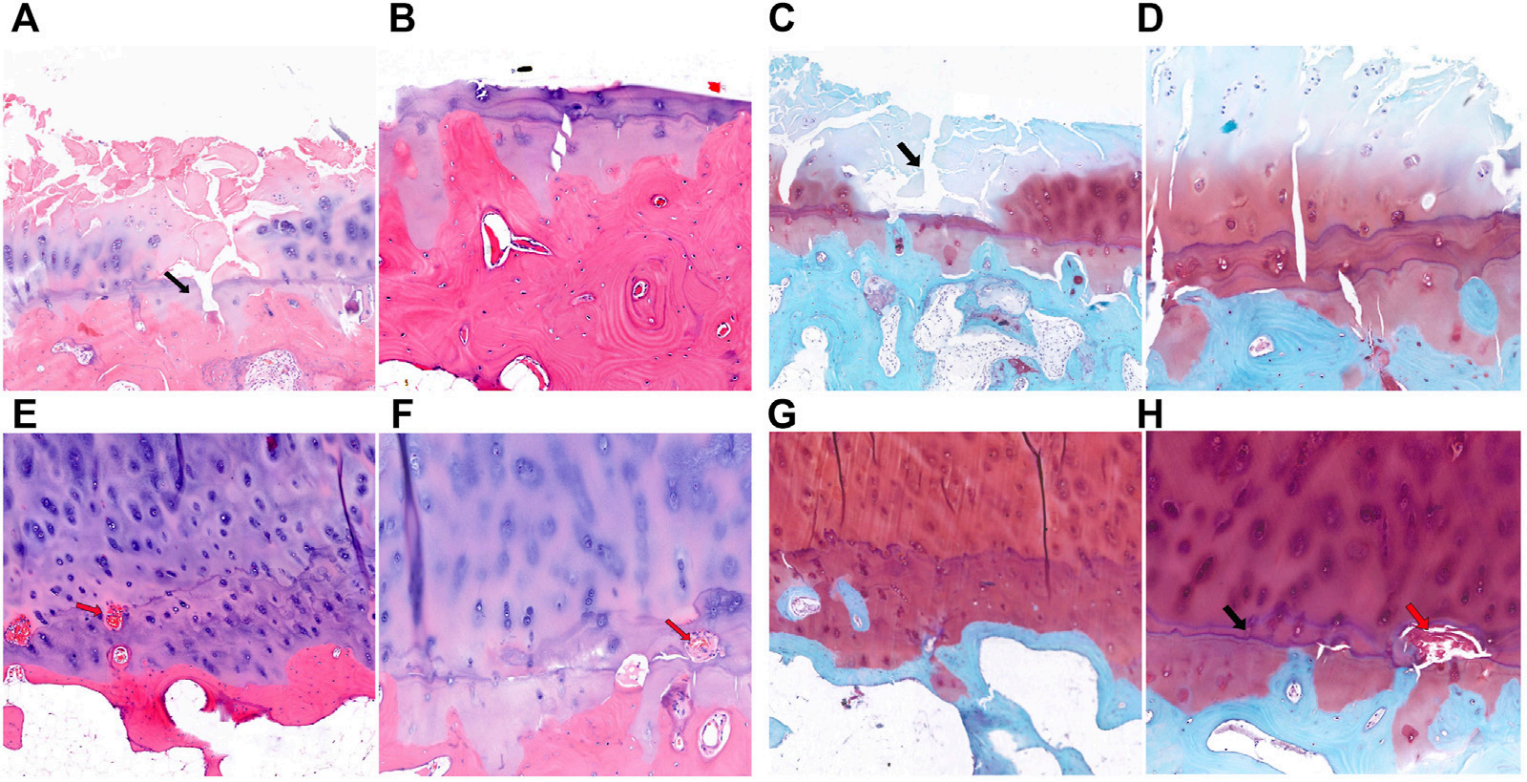
HE and safranin O-fast green staining of cartilage and subchondral bone of the tibial plateau in OA patients. **(A,B)**, HE staining of the medial tibial plateau. The black arrow shows a blurred tidemark and indistinguishable calcified layer (magnification: **(A)**, ×5; **(B)**, ×10). **(C,D)**, Safranin O-fast green staining of the medial tibial plateau. The black arrow shows a defect of cartilage (magnification: **(C)**, ×5; **(D)**, ×10). **(E,F)**, HE staining of the lateral tibial plateau. The red arrow shows vascular invasion (magnification: **(E)**, ×5; **(F)**, ×10). **(G,H)**, Safranin O-fast green staining of the lateral tibial plateau. The black arrow shows tidemark replication, and the red arrow shows vascular invasion (magnification: **(G)**, ×5; **(H)**, ×10).

Similar to KBD, in some sections of the medial tibial plateau in OA patients, almost the entire layer of cartilage was missing, and only a few chondrocytes were visible. The tidemark was blurred, interrupted, or absent, and the calcified layer was indistinguishable (Figures 3A–D). In some sections of the lateral tibial plateau, there were defects in the superficial layer of cartilage, tiny fissures through the deep layer of cartilage, duplicated tidemarks, and vascular invasion in the calcified area (Figures 3E,F). The subchondral bone of the tibial plateau in OA patients also showed increased thickness and sclerosis.

In addition, we examined potential collagen changes in tibial plateau subchondral bone by picrosirius red staining (Supplementary Figure S1). The KBD tibial plateau subchondral bone showed yellow-red staining of type I collagen with strong birefringence, and there was no significant difference between the medial and lateral tibial plateau samples. The OA tibial plateau subchondral bone also showed yellow-red staining of type I collagen, but with some green staining in the middle, showing strong birefringence, and the thickness in the medial tibial plateau was significantly higher than that in the lateral side.

### Identification and analysis of differentially expressed genes

**FIGURE 4:**
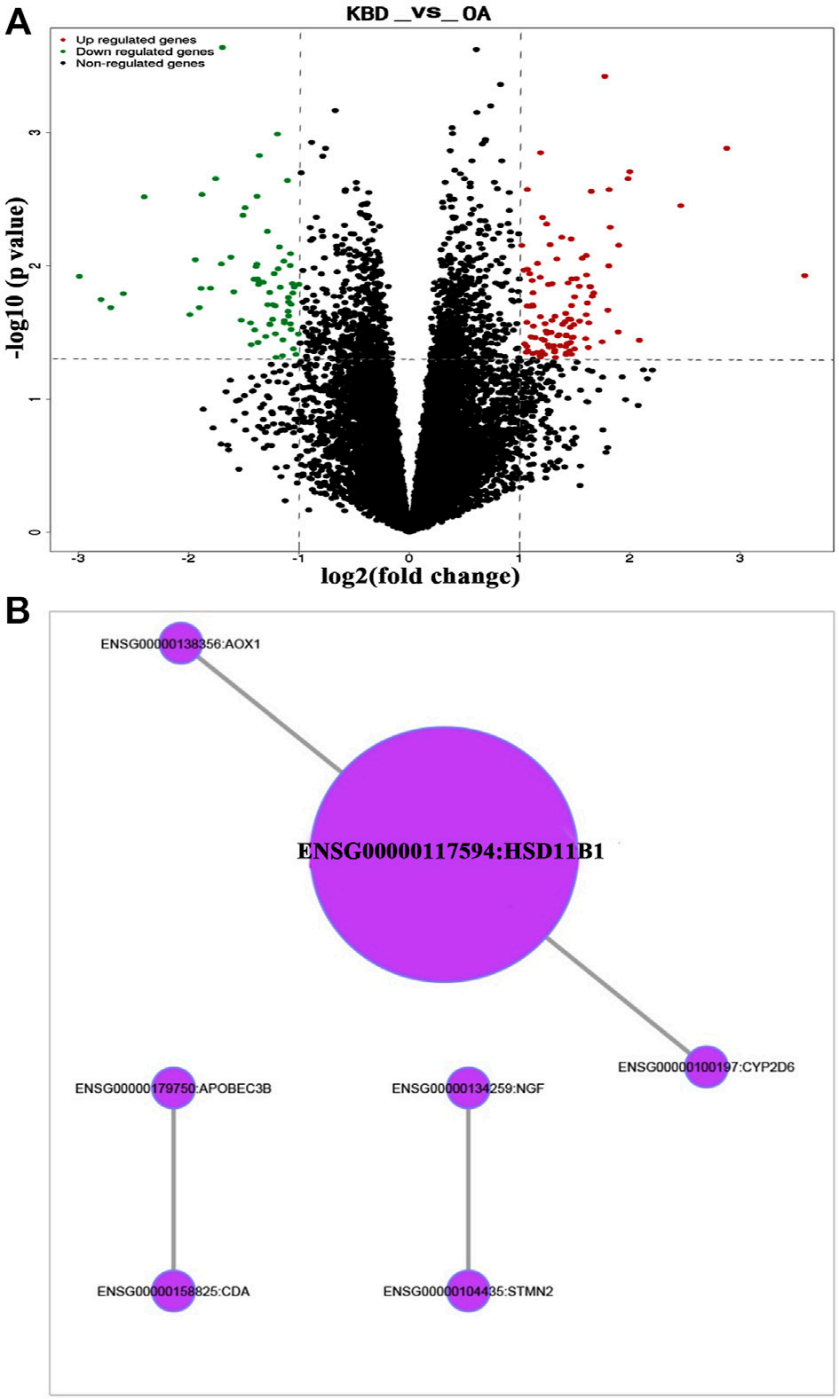
RNA sequencing analysis of differentially expressed genes in subchondral bone between patients with KBD and OA. **(A)**, Volcano plot filtering of the differentially expressed genes. The vertical lines represent–log10 (*p* value), and the horizontal line represents log2 (fold change). The red and green points in the plot represent the up- and downregulated genes with statistical significance, respectively. **(B)**, Protein-protein interaction network of the differentially expressed genes in subchondral bone between patients with KBD and OA.

RNA-sequencing generated 46.1 to 49.2 million raw reads among the 18 samples, of which 96.5%–97.3% were clean reads. A total of 56,375 unigenes were assembled, with an average transcript length of 1,721 bp. Out of 56,375 unigenes, 38,930 (69.1%) were successfully annotated based on five databases. Under the thresholds of |log2-fold change| ≥ 1 and P value ≤0.05, we identified 179 DEGs in the subchondral bone between KBD and OA. Of these, 109 genes were upregulated and 70 were downregulated in KBD subchondral bone compared to OA subchondral bone (Figure 4A; Supplementary Table S3). Hierarchical cluster analysis confirmed that the expression of DEGs in subchondral bone from KBD patients could be clearly distinguished from that from OA patients (Supplementary Figure S2).

GO and KEGG pathway enrichment analyses were performed on DEGs. Using the corrected P value ≤0.05 as the threshold, the 179 DEGs that could be categorized into 127 GO functional groups, which included 117 biological processes, nine molecular functions, and one cellular component (Supplementary Table S4). KEGG enrichment analysis showed that the 179 DEGs were associated with 5 metabolic and signal transduction pathways, including drug metabolism-cytochrome P450, metabolism of xenobiotics by cytochrome P450, vitamin B6 metabolism, MAPK signaling pathway, and glycosphingolipid biosynthesis. A classification of the KEGG pathways based on different categories of the DEGs is shown in Supplementary Figure S3. In addition, the protein-protein interaction network of the DEGs revealed seven central node genes, namely *HSD11B1*, *CYP2D6*, *CDA*, *APOBEC3B*, *AOX1*, *STMN2*, and *NGF* (Figure 4B).

### Identification and validation of differentially expressed miRNAs

**FIGURE 5:**
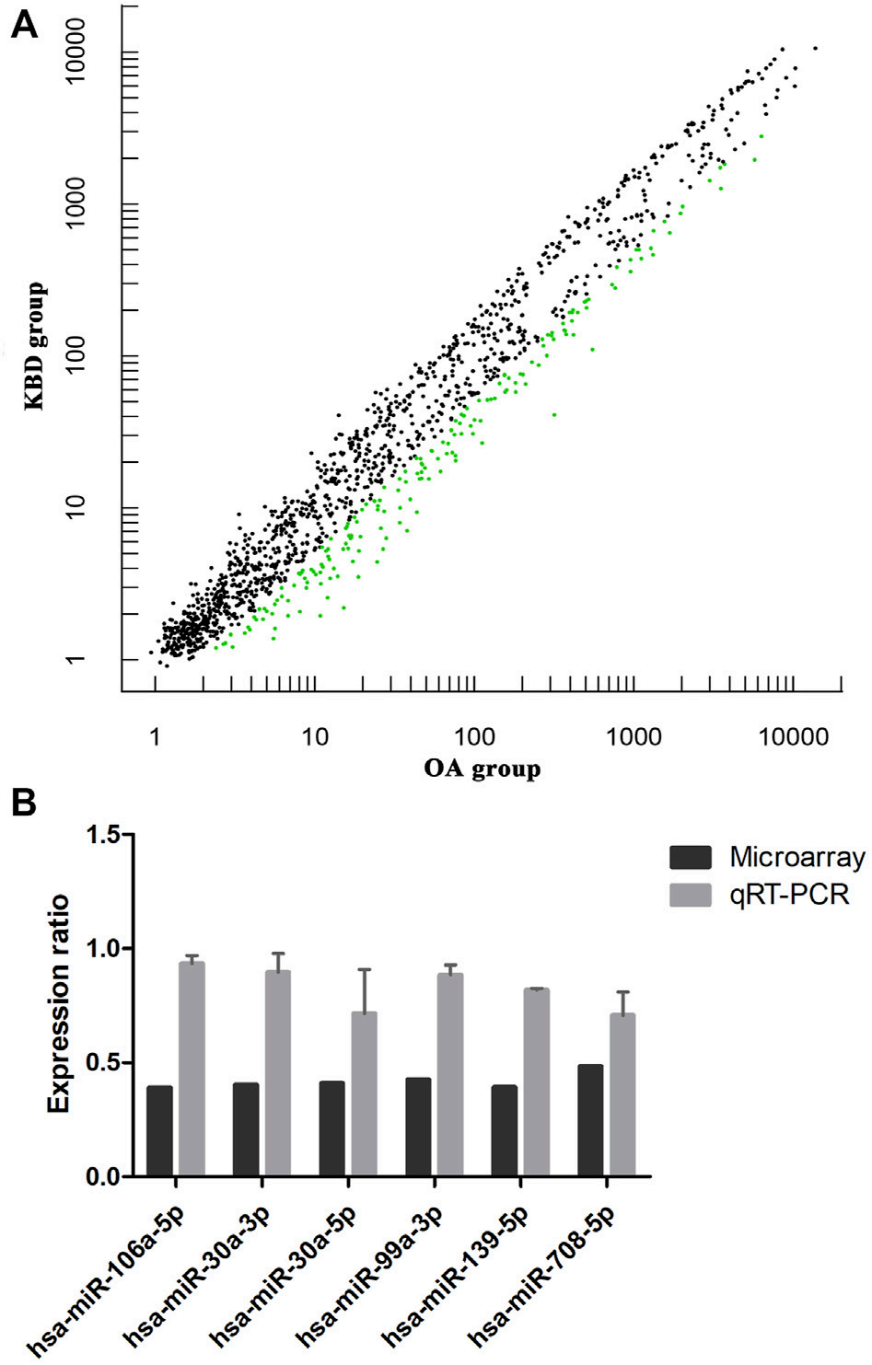
Microarray profiling analysis of differentially expressed miRNAs in subchondral bone between patients with KBD and OA. **(A)**, Scatter plot filtering of the differentially expressed miRNAs. The green points represent significantly downregulated miRNAs in the KBD versus OA group, and the black points are genes with no significant differences. **(B)**, Validation of six differentially expressed miRNAs by qRT-PCR.

miRNA microarray analysis was performed to identify DEMs. Hierarchical cluster analysis revealed that the miRNA expression patterns were significantly different between the two groups (Supplementary Figure S4). Based on a twofold change cutoff (q value ≤0.05), a total of 124 DEMs were identified, and all of them were downregulated in the subchondral bone of KBD patients compared to OA patients (Figure 5A; Supplementary Table S5). To validate our microarray findings, six DEMs (hsa-miR-30a-3p, hsa-miR-30a-5p, hsa-miR-99a-3p, hsa-miR-106a-5p, hsa-miR-139-5p, and hsa-miR-708-5p) were selected for qRT-PCR. Consistent with the microarray data, the qRT-PCR results also showed downregulation of the six miRNAs in the subchondral bone of KBD compared to OA (Figure 5B).

To obtain the potential targets for all DEMs, computational predictions were carried out using twelve different prediction programs. As expected, most of these miRNAs potentially regulate hundreds of targeted genes. To limit the total number of false-positives, the targets were considered putative candidates when six or more of these programs were predicted simultaneously. A list of the potential miRNA-targeted genes is shown in Supplementary Table S6.

### Integration analysis of DEMs and targeted DEGs

**FIGURE 6:**
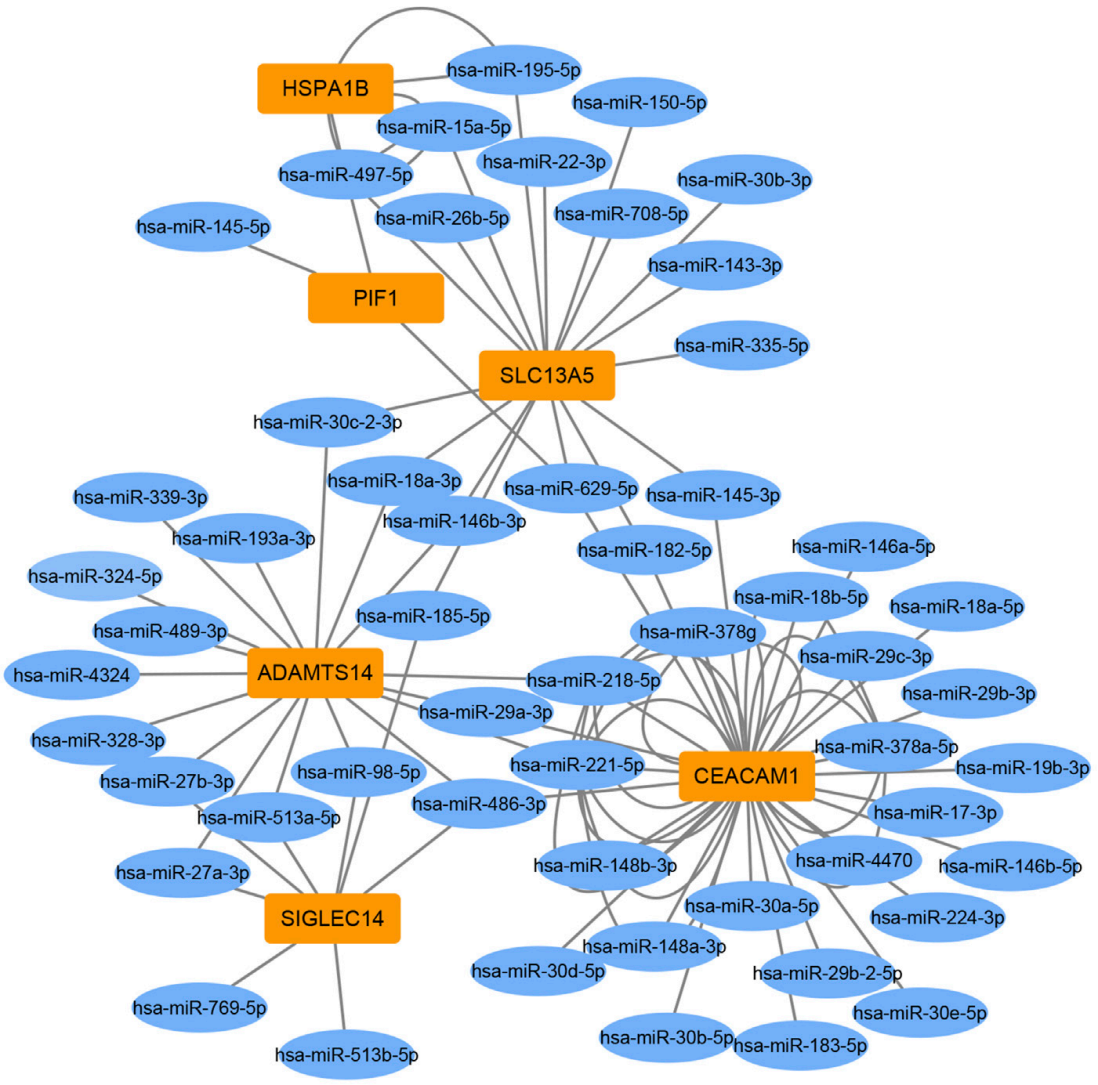
Construction of miRNA-target gene interaction networks.

To identify miRNA-target gene interaction pairs and key DEGs in the subchondral bone between patients with KBD and OA, integrated analysis was conducted to make correlations following the criterion of reverse regulation of a miRNA and a corresponding mRNA. We used the 124 downregulated miRNAs to correlate with the 106 upregulated DEGs, and the results showed that 76 miRNA-mRNA pairs were negatively correlated, which included of 48 miRNAs and 6 targeted genes (Supplementary Table S7). The miRNA-target gene interaction networks revealed that *ADAMTS14*, *SLC13A5*, and *CEACAM1* may be important DEGs in subchondral bone between patients with KBD and OA (Figure 6).

### Verification of the key DEGs by immunohistochemistry

**FIGURE 7:**
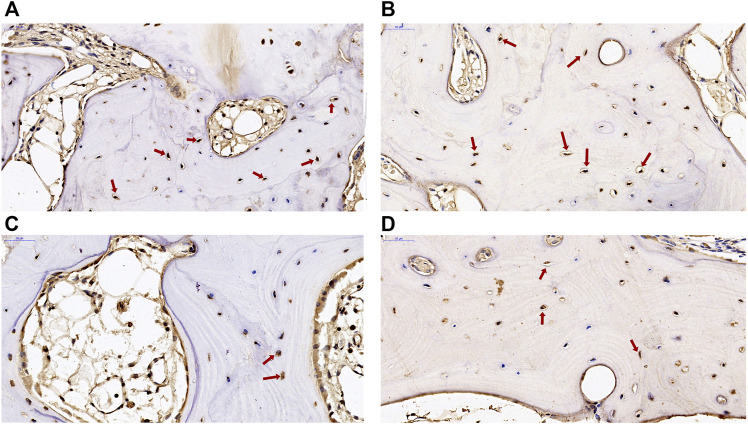
Immunohistochemical staining of *ADAMTS14* and *SLC13A5* in subchondral bone from patients with KBD and OA. **(A,B)**, the expression of *ADAMTS14* in subchondral bone from patients with KBD and OA, respectively (magnification: ×40). **(C,D)**, The expression of SLC13A5 in subchondral bone from patients with KBD and OA, respectively (magnification: ×40). The red arrows represent the positively stained cells.

Two DEGs of *ADAMTS14* and *SLC13A5* were selected for immunohistochemical verification in this study. Positive staining of the two genes was found in osteocytes and osteoblasts in subchondral bone from patients with KBD and OA. As shown in Figure 7, the positive expression rate of *ADAMTS14* and *SLC13A5* in subchondral bone of KBD patients were higher than those of OA patients. The results confirmed that the two genes were differentially expressed in the subchondral bone between patients with KBD and OA.

## Discussion

Although the onset of KBD occurs primarily in childhood, as the disease progresses, adult patients with KBD often develop severe cartilage damage to multiple joints similar to patients with OA. However, KBD and OA still present some different clinical features and should be differentially diagnosed (Li Y. et al., 2013). Previous studies have better distinguished the difference in cartilage pathology between KBD and OA, and the findings of the present study revealed the main histopathological changes, some key DEGs, and pathways of the subchondral bone between the two diseases.

OA is now considered a complex condition affecting the whole joint including articular cartilage, subchondral bone, ligaments, and synovium. Histopathological staining revealed many alterations in the osteochondral plate and subchondral bone of OA, including fragmentation of the articular cartilage, duplication and advancement of the tidemark, expansion and vascular invasion of the calcified cartilage zone, and thickening of the subchondral bone plate (Martel-Pelletier et al., 2016). The results of this study confirmed these structural changes in the OA tibial plateau. Interestingly, except for the typical deep chondrocyte necrosis in KBD cartilage, our study revealed that histopathological changes in the articular cartilage and subchondral bone of KBD were similar to those of OA. Our findings indicate that KBD presents some pathological changes in secondary OA. Therefore, it is difficult to distinguish the histopathological changes in subchondral bone between KBD and OA.

Because all of the OA patients in this study were in an advanced stage and most of them exhibited varus deformities of the knee joints, they had more severe damage to the articular cartilage in the medial tibial plateau than in the lateral tibial plateau. A study showed that as cartilage loss occurred in the medial tibia, the bone volume fraction and trabecular thickness increased in the medial tibia and decreased in the lateral tibia (Chiba et al., 2012). Our results demonstrated that the thickness of the subchondral bone in the medial tibial plateau was increased compared to the lateral side in both OA and KBD patients. Mechanical loading plays a critical role in the pathogenesis of OA (Hunt et al., 2020). The difference in damage degree between the medial and lateral tibia in this study may be due to a shift in loading on the medial joint and a decrease in loading on the lateral joint as the knee joint varus deformed in OA patients. The medial and lateral tibias of adult KBD patients exhibit similar characteristics as OA, which suggests that mechanical loading also plays an important role in the progression of KBD.

We further compared the transcriptomics and miRNAs to explore differences in gene expression patterns of subchondral bone between patients with KBD and OA and to identify disease-related target genes. The DEG screening from RNA sequencing in this study showed that there were 179 DEGs in subchondral bone between KBD and OA, with a total of 109 upregulated genes and 70 downregulated genes. miRNA array profiling showed that 124 miRNAs were significantly down-regulated in KBD subchondral bone compared with OA subchondral bone. These results indicate that the gene expression patterns as well as the pathological mechanisms are different in subchondral bone between KBD and OA.

A previous genome profiling study identified 972 DEGs in OA subchondral bone between the medial tibial plateau (significant degeneration region) and the lateral tibial plateau (minimal degeneration region), with a total of 420 upregulated genes and 552 downregulated genes (Chou et al., 2013). By comparing the 972 DEGs sets and the 179 DEGs (KBD vs. OA) in this study, a total of 2 co-upregulated DEGs (*ADAMTS14*, *SLC13A5*) and 2 co-downregulated DEGs (*HSD11B1*, *BMP5*) were identified. The findings showed that the four DEGs were more significantly modified in KBD subchondral bone than that in OA subchondral bone, suggesting that the subchondral osteosclerosis may be more severe in KBD than in OA, but quantitative studies are still needed to further confirm this. In addition, a miRNA profiling study identified a set of 30 DEMs between the sclerotic (medial tibial plateau) and non-sclerotic (lateral tibial plateau) OA subchondral bone (Prasadam et al., 2016). In which, ten of them (has-miR-17-5p, has-miR-20b-5p, has-miR-30a-3p, has-miR-30a-5p, has-miR-30d-5p, has-miR-30e-5p, has-miR-99a-3p, has-miR-106a-5p, has-miR-148a-3p and has-miR-708-5p) were also identified as DEMs between KBD and OA subchondral bone in this study.

Enrichment analysis of the screened DEGs showed that these genes were involved in numerous GO terms and signaling pathways. Among them, several biological processes were involved in extrinsic apoptosis, such as the extrinsic apoptotic signaling pathway and regulation of extrinsic apoptotic signaling pathway. Chondrocyte necroptosis and apoptosis were observed in the cartilage of KBD (Zhang et al., 2018), suggesting possible molecular crosstalk of apoptotic signaling between the subchondral bone and cartilage in KBD. The mitogen-activated protein kinase (MAPK) signaling pathway was significantly enriched by KEGG analysis in this study. MAPK signaling has important roles in osteogenesis and bone homeostasis, and is involved in subchondral bone change in OA progression (Prasadam et al., 2010). In addition, some studies have shown that MAPK signaling is a crucial pathway for subchondral bone treatment in OA (Yajun et al., 2021; Zhang et al., 2022).

Protein-protein interaction analysis revealed that *HSD11B1*, an important central node gene, was downregulated in KBD subchondral bone compared with OA subchondral bone. Glucocorticoids have vital effects on bone through multiple pathways. Glucocorticoid regulation is crucially controlled by 11β-hydroxysteroid dehydrogenase (11β-HSD) isozymes. 11β-HSD1 (*HSD11B1*) is the major glucocorticoid-modifying enzyme in bone that is expressed in human osteoblasts *in vivo* and affects osteoblastic proliferation and differentiation (Tomlinson et al., 2004). A study showed an association of *HSD11B1* polymorphisms with osteoporosis in postmenopausal women, suggesting the possible involvement of this protein in bone metabolism (Hwang et al., 2009). Localized osteoporotic changes were also observed in KBD, which may be related to 11β-HSD1 enzyme activity in osteoblasts of KBD, but further studies are needed to confirm this hypothesis.

The present study identified several key DEGs in subchondral bone between KBD and OA by integrated analysis. We found that *ADAMTS14* and *SLC13A5* were more significantly positively expressed in KBD subchondral bone than in OA subchondral bone. *ADAMTS14* is a member of the metalloproteinase family that is involved in collagen biosynthesis as a procollagen propeptidase. *ADAMTS14* was shown to be significantly upregulated in OA cartilage, reflecting the increase in collagen synthesis by chondrocytes and probably in an attempt to repair the cartilage matrix of OA cartilage (Davidson et al., 2006). In addition, a study showed that *ADAMTS14* gene polymorphism was associated with knee OA, and the GG genotype increased the risk of knee OA in the Chinese Han population (Ma et al., 2018). However, the relationship between the *ADAMTS14* gene and bone or subchondral bone lesions remains unclear. In addition, as a vital membrane transporter for citrate, the expression of *SLC13A5* was upregulated in bone matrix formation (Mantila Roosa et al., 2011), and *SLC13A5* deficiency led to decreased bone mineral density and impaired bone formation in mice (Irizarry et al., 2017). Our findings suggest that the upregulation of *SLC13A5* expression may be involved in the sclerosis of KBD subchondral bone.

Differential diagnosis of diseases is to study different etiology and pathogenesis of diseases from similar symptoms, which is the basis and key step of disease treatment. The findings of this study provided important candidate molecular markers for the differential diagnosis of pathological changes of subchondral bone between KBD and OA. However, the main limitation of this study is that normal subchondral bone specimens were not collected in the study as controls, which led to significant difficulties in explaining the mechanism of the identified DEGs in the pathogenesis of KBD and OA. Another limitation is that this study only examined the pathological changes by histological staining and did not quantitatively analyze the structural parameters of cartilage and subchondral bone in both groups.

In conclusion, we first comparatively demonstrated the histopathological changes, transcriptomics, and miRNAs of subchondral bone in the tibial plateau between KBD and OA. Although subchondral bone samples of KBD and OA exhibited similar histopathological characteristics, they had different gene expression patterns by RNA-sequencing and miRNA microarray analysis, suggesting different pathological mechanisms of subchondral bone between the two diseases. The integrated analysis further revealed several genes, such as *ADAMTS14*, *SLC13A5*, and *CEACAM1*, that may be crucial DEGs in subchondral bone between KBD and OA, suggesting that these genes could serve as potential differential diagnostic biomarkers for subchondral bone lesions in KBD and OA. The findings of this study provide valuable information for further study to clarify pathological changes in subchondral bone in KBD and OA.

## Data Availability

The datasets presented in this study can be found in online repositories. The names of the repository/repositories and accession number(s) can be found below: https://ngdc.cncb.ac.cn/bioproject/browse/PRJCA009526.
