# Supplementary material for: Comprehensive comparative analysis of histopathology and gene expression in subchondral bone between kashin-beck disease and primary osteoarthritis
Source: Front Genet. 2022 Jul 18;13:942326. doi: 10.3389/fgene.2022.942326 (PMC9339956; doi:10.3389/fgene.2022.942326)
Supplement: Supplementary file 1 [file DataSheet1.PDF]

# *Supplementary Material*

## 1 Supplementary Figures

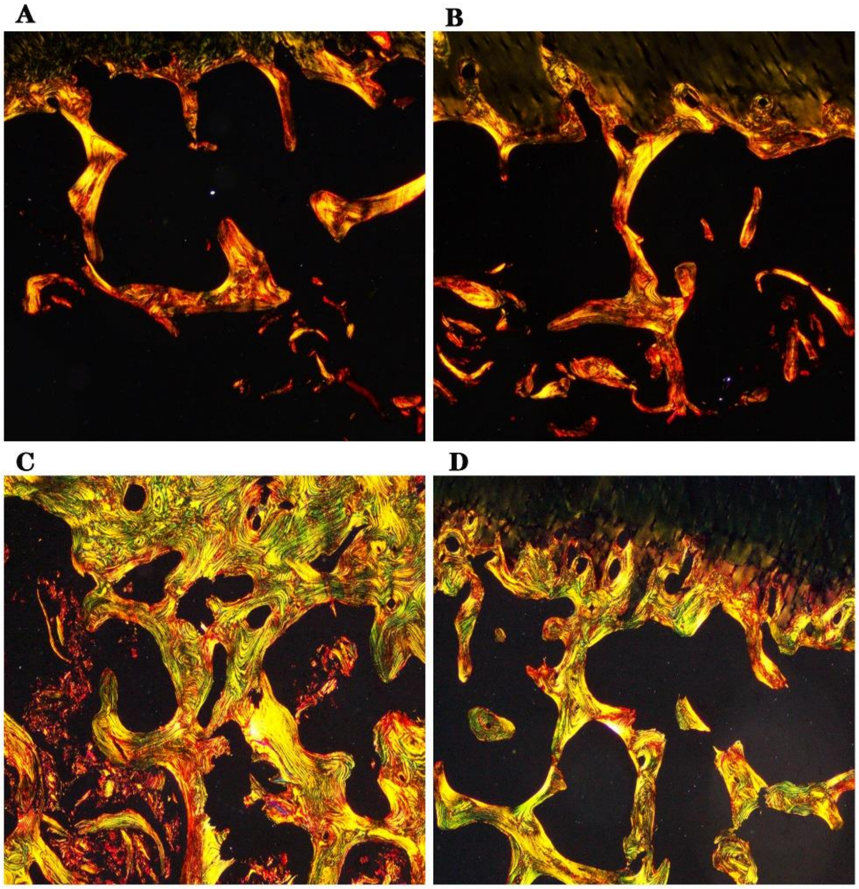


**Supplementary Figure S1.** Picrosirius red staining of tibial plateau subchondral bone in patients with KBD and OA. A-B, The medial and lateral tibial plateau in KBD patients, respectively (magnification: 40×). C-D, The medial and lateral tibial plateau in OA patients, respectively (magnification: 40×).

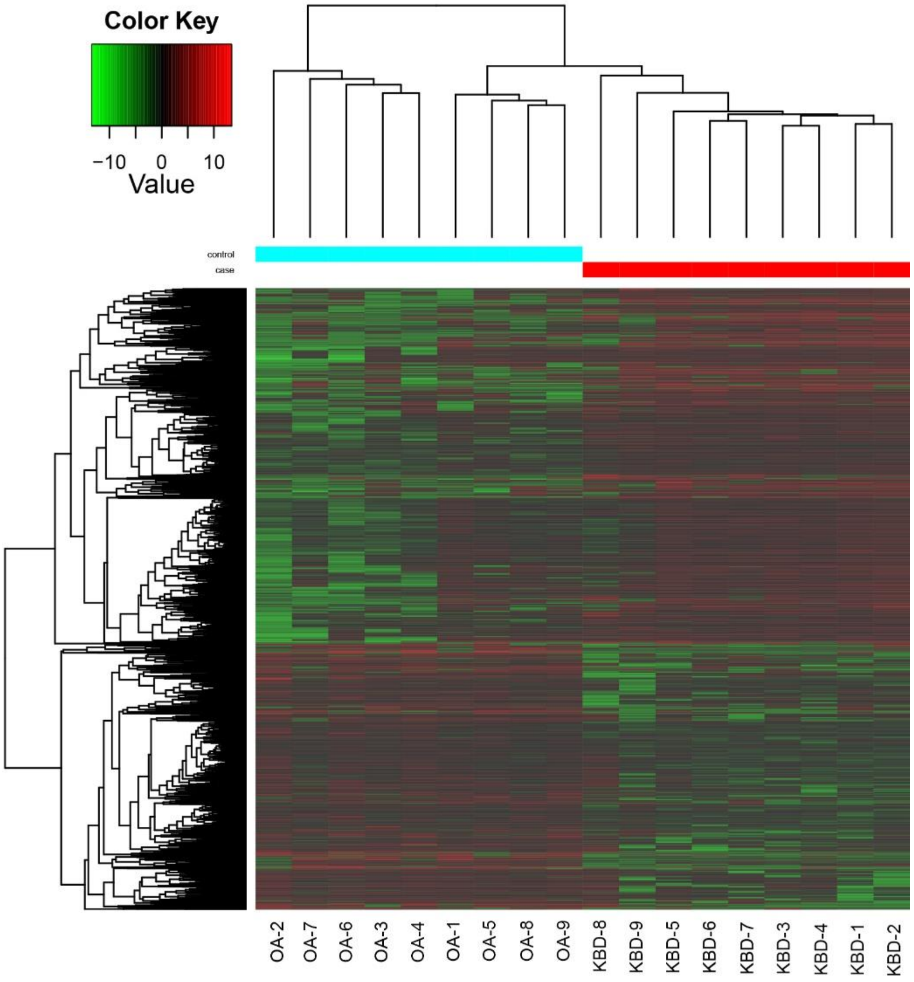


**Supplementary Figure S2.** Hierarchical cluster analysis of differentially expressed genes in the subchondral bone between patients with KBD and OA. Different colored regions represent different clustering grouping information, and gene expression patterns within the same group are similar and may be involved in the same biological processes.

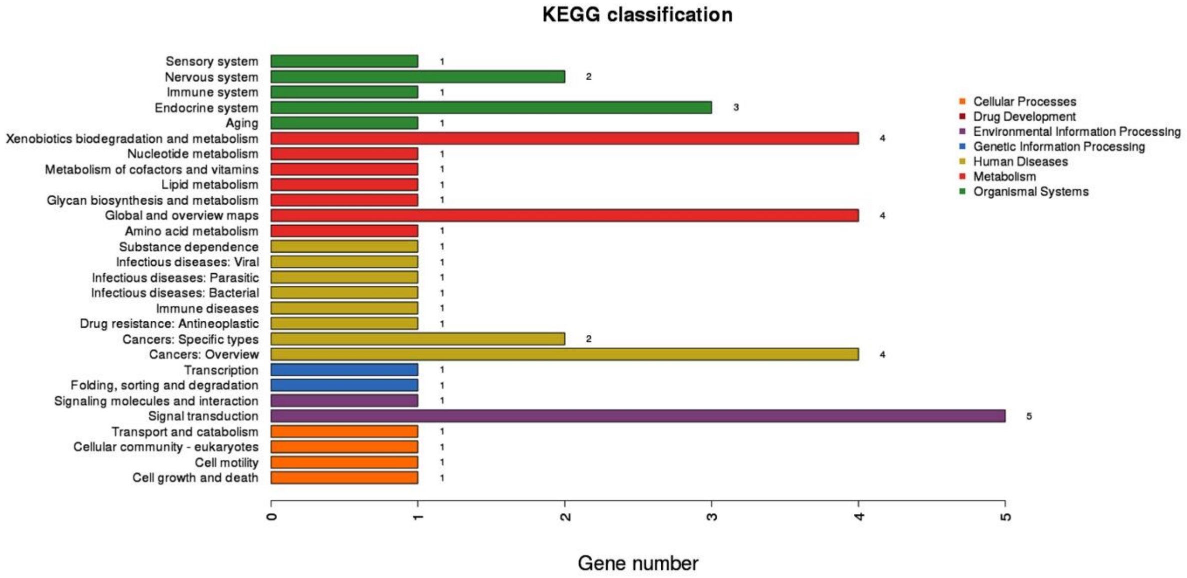


**Supplementary Figure S3.** KEGG classification based on different categories of the DEGs in the subchondral bone between patients with KBD and OA. The vertical line is the name of the KEGG pathway, and the horizontal line represents the number of genes annotated to the pathway.

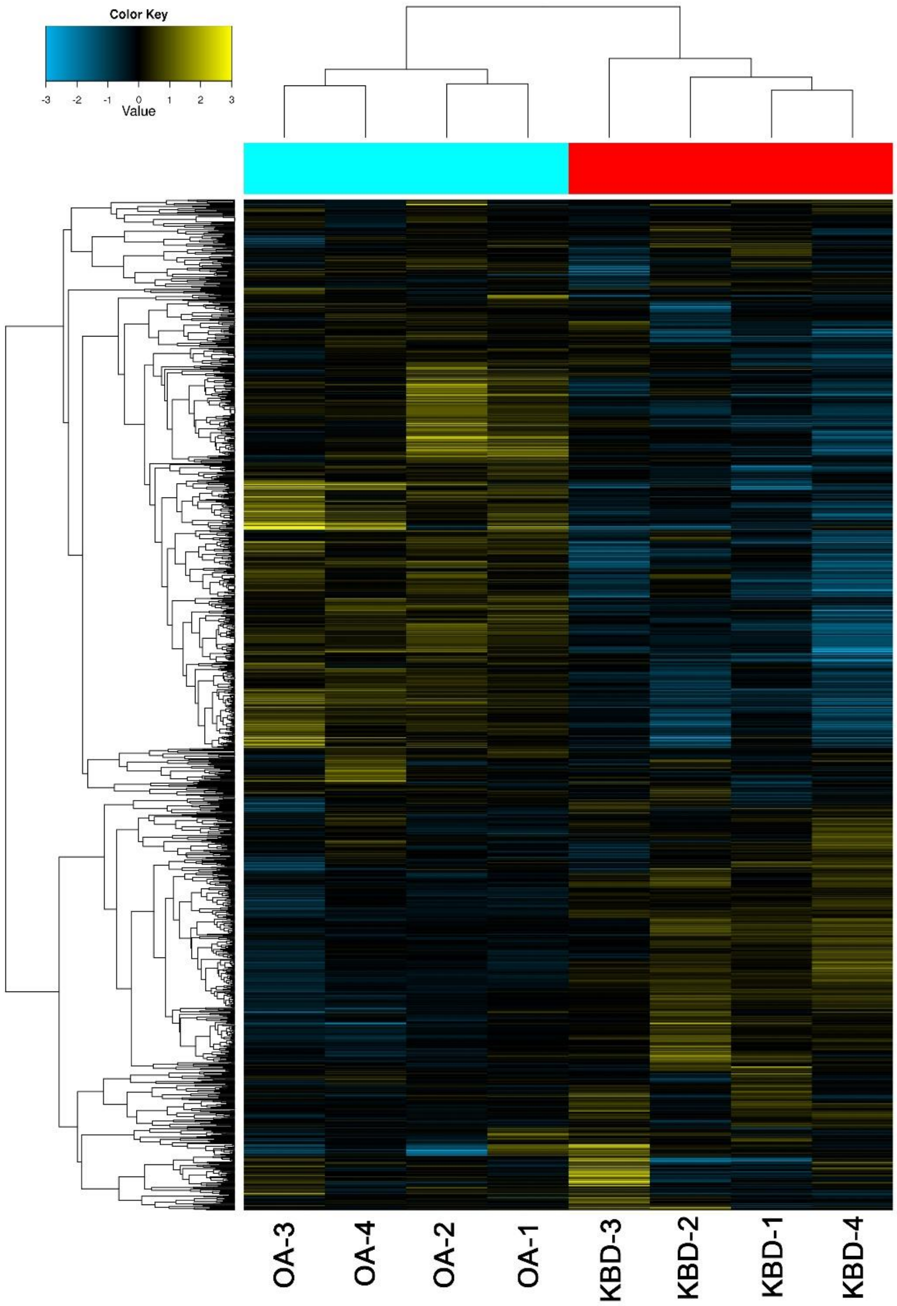


**Supplementary Figure S4.** Hierarchical cluster analysis showing the differential expression pattern of miRNAs in the subchondral bone between patients with KBD and OA.

## 2 Supplementary Tables

**Supplementary Table S1**. The basic characteristics of included patients with KBD and OA.

| Assay | No. | KBD | | | OA | | |
|---|---|---|---|---|---|---|---|
| | | Gender | Age (years) | K-L grade | Gender | Age (years) | K-L grade |
| Histological assay | 1 | Female | 65 | IV | Female | 66 | IV |
| | 2 | Female | 74 | IV | Female | 68 | IV |
| | 3 | Female | 64 | IV | Female | 64 | IV |
| | 4 | Male | 59 | IV | Male | 68 | IV |
| | 5 | Male | 61 | IV | Male | 72 | IV |
| mRNA sequencing | 6 | Male | 59 | IV | Female | 65 | IV |
| | 7 | Female | 54 | IV | Male | 70 | IV |
| | 8 | Female | 57 | IV | Female | 62 | IV |
| | 9 | Female | 60 | IV | Female | 62 | IV |
| | 10 | Female | 52 | IV | Female | 66 | IV |
| | 11 | Female | 67 | IV | Female | 75 | IV |
| | 12 | Male | 74 | IV | Female | 62 | IV |
| | 13 | Male | 65 | IV | Male | 70 | IV |
| | 14 | Female | 64 | IV | Female | 69 | IV |
| miRNA array profiling | 15 | Female | 58 | IV | Female | 56 | IV |
| | 16 | Male | 59 | IV | Female | 58 | IV |
| | 17 | Male | 60 | IV | Male | 62 | IV |
| | 18 | Female | 54 | IV | Male | 60 | IV |

**Supplementary Table S2.** The miRNA primers used in qRT-PCR

| Gene name | | Primer sequence |
|---|---|---|
| Has-miR-106a-5p | RT | GTCGTATCCAGTGCAGGGTCCGAGGTATTCGCACTGGATACGACctacct |
| | AS | CGGCAAAAGTGCTTACAGT |
| Has-miR-30a-3p | RT | GTCGTATCCAGTGCAGGGTCCGAGGTATTCGCACTGGATACGACgctgca |
| | AS | GGGCTTTCAGTCGGATG |
| Has-miR-30a-5p | RT | GTCGTATCCAGTGCAGGGTCCGAGGTATTCGCACTGGATACGACcttcca |
| | AS | CGGTGTAAACATCCTCGACT |
| Has-miR-99a-3p | RT | GTCGTATCCAGTGCAGGGTCCGAGGTATTCGCACTGGATACGACcagacc |
| | AS | CAAGCTCGCTTCTATGGG |
| hsa-miR-139-5p | RT | GTCGTATCCAGTGCAGGGTCCGAGGTATTCGCACTGGATACGACactgga |
| | AS | TCTACAGTGCACGTGTCTCC |
| hsa-miR-708-5p | RT | GTCGTATCCAGTGCAGGGTCCGAGGTATTCGCACTGGATACGACcccagc |
| | AS | CTCTGAAGGAGCTTACAATCTAG |

**Supplementary Table S3.** The differentially expressed genes in subchondral bone from patients with KBD versus OA by RNA-sequencing.

| ID | symbol | logFC | *P*-value |
|---|---|---|---|
| **Upregulated genes** | | | |
| ENSG00000233695 | *GAS6-AS1* | 1.07 | 0.003 |
| ENSG00000137877 | *SPTBN5* | 1.21 | 0.004 |
| ENSG00000243302 | *AC018638.4* | 1.06 | 0.011 |
| ENSG00000247982 | *LINC00926* | 1.02 | 0.007 |
| ENSG00000201861 | *RNA5SP298* | 1.77 | 0.000 |
| ENSG00000100197 | *CYP2D6* | 1.24 | 0.005 |
| ENSG00000140451 | *PIF1* | 1.07 | 0.011 |
| ENSG00000273837 | *AC018755.4* | 2.88 | 0.001 |
| ENSG00000234353 | *AP000346.2* | 1.19 | 0.001 |
| ENSG00000175170 | *FAM182B* | 1.04 | 0.011 |
| ENSG00000226686 | *LINC01535* | 1.65 | 0.003 |
| ENSG00000138316 | *ADAMTS14* | 1.07 | 0.027 |
| ENSG00000141485 | *SLC13A5* | 1.60 | 0.023 |
| ENSG00000227370 | *AC254562.1* | 2.00 | 0.002 |
| ENSG00000173597 | *SULT1B1* | 1.29 | 0.026 |
| ENSG00000272512 | *AL645608.8* | 1.32 | 0.024 |
| ENSG00000230847 | *AC044797.1* | 2.46 | 0.004 |
| ENSG00000203286 | *RF00017* | 1.81 | 0.003 |
| ENSG00000243433 | *AC010973.1* | 1.98 | 0.002 |
| ENSG00000260941 | *LINC00622* | 1.09 | 0.011 |
| ENSG00000279382 | *AC018665.1* | 1.10 | 0.020 |
| ENSG00000236871 | *LINC00106* | 1.12 | 0.020 |
| ENSG00000285336 | *AC108734.4* | 1.64 | 0.014 |
| ENSG00000267986 | *AC130469.1* | 1.57 | 0.009 |
| ENSG00000240823 | *RN7SL23P* | 1.82 | 0.005 |
| ENSG00000111215 | *PRR4* | 1.28 | 0.007 |
| ENSG00000204388 | *HSPA1B* | 1.07 | 0.043 |
| ENSG00000279713 | *AC080038.3* | 1.46 | 0.025 |
| ENSG00000079385 | *CEACAM1* | 1.12 | 0.028 |
| ENSG00000258904 | *AL157871.5* | 1.90 | 0.007 |
| ENSG00000122136 | *OBP2A* | 1.64 | 0.014 |
| ENSG00000260367 | *AC109460.1* | 1.16 | 0.010 |
| ENSG00000227495 | *AC004771.1* | 1.38 | 0.006 |
| ENSG00000201512 | *SNORA71C* | 1.60 | 0.008 |
| ENSG00000262903 | *AC027796.4* | 1.12 | 0.016 |
| ENSG00000254415 | *SIGLEC14* | 3.58 | 0.012 |
| ENSG00000269997 | *AC068790.3* | 1.47 | 0.006 |
| ENSG00000273444 | *AC006023.2* | 1.19 | 0.012 |
| ENSG00000232725 | *U52111.1* | 1.51 | 0.012 |
| ENSG00000262434 | *AC087392.4* | 1.81 | 0.010 |
| ENSG00000249852 | *AC145676.1* | 1.12 | 0.020 |
| ENSG00000254701 | *AC138866.2* | 1.47 | 0.013 |

| | | | |
|---|---|---|---|
| ENSG00000237082 | *COX5BP6* | 1.34 | 0.009 |
| ENSG00000277744 | *AC011462.4* | 1.66 | 0.017 |
| ENSG00000265791 | *AC127024.4* | 1.11 | 0.013 |
| ENSG00000264188 | *AC106037.1* | 1.61 | 0.012 |
| ENSG00000264384 | *RN7SL431P* | 1.44 | 0.013 |
| ENSG00000228242 | *AC093495.1* | 1.06 | 0.040 |
| ENSG00000279759 | *AC118344.2* | 1.16 | 0.045 |
| ENSG00000269951 | *AC090181.2* | 1.26 | 0.014 |
| ENSG00000276718 | *AC005840.4* | 1.67 | 0.016 |
| ENSG00000212371 | *RF00404* | 1.50 | 0.017 |
| ENSG00000280067 | *AC023818.1* | 1.24 | 0.031 |
| ENSG00000273240 | *AC013468.1* | 1.54 | 0.014 |
| ENSG00000204044 | *SLC12A5-AS1* | 1.47 | 0.035 |
| ENSG00000229901 | *AC093151.2* | 1.29 | 0.014 |
| ENSG00000261786 | *AC006058.1* | 1.20 | 0.044 |
| ENSG00000223374 | *AC005104.1* | 1.10 | 0.034 |
| ENSG00000133136 | *GNG5P2* | 1.19 | 0.024 |
| ENSG00000279837 | *AC112694.2* | 1.41 | 0.026 |
| ENSG00000241217 | *RN7SL809P* | 1.47 | 0.020 |
| ENSG00000274767 | *AC243829.1* | 1.32 | 0.044 |
| ENSG00000227782 | *AC002553.1* | 1.20 | 0.036 |
| ENSG00000215493 | *AC007731.2* | 1.42 | 0.040 |
| ENSG00000179750 | *APOBEC3B* | 2.09 | 0.036 |
| ENSG00000275413 | *AC002553.2* | 1.23 | 0.027 |
| ENSG00000273055 | *AC005046.1* | 1.43 | 0.038 |
| ENSG00000243854 | *RN7SL67P* | 1.80 | 0.022 |
| ENSG00000273338 | *AC103591.3* | 1.61 | 0.019 |
| ENSG00000263264 | *AC119396.1* | 1.44 | 0.025 |
| ENSG00000276505 | *AP000892.2* | 1.07 | 0.020 |
| ENSG00000268279 | *AC090004.1* | 1.04 | 0.039 |
| ENSG00000255142 | *AP006621.2* | 1.42 | 0.034 |
| ENSG00000273096 | *AL021707.8* | 1.42 | 0.023 |
| ENSG00000230928 | *AL139241.1* | 1.23 | 0.036 |
| ENSG00000199331 | *RF00019* | 1.29 | 0.032 |
| ENSG00000268670 | *AC016586.1* | 1.54 | 0.026 |
| ENSG00000211599 | *IGKV5-2* | 1.89 | 0.031 |
| ENSG00000228206 | *AC016717.1* | 1.63 | 0.027 |
| ENSG00000225964 | *NRIR* | 1.27 | 0.041 |
| ENSG00000232546 | *AC027644.1* | 1.36 | 0.033 |
| ENSG00000225920 | *RIMKLBP2* | 1.37 | 0.040 |
| ENSG00000255176 | *AP000941.1* | 1.50 | 0.042 |
| ENSG00000226871 | *AC135178.1* | 1.46 | 0.029 |
| ENSG00000264520 | *AC005154.5* | 1.49 | 0.028 |
| ENSG00000257509 | *AC073487.1* | 1.44 | 0.039 |
| ENSG00000235408 | *SNORA71B* | 1.39 | 0.028 |
| ENSG00000178440 | *LINC00843* | 1.26 | 0.039 |

| | | | |
|---|---|---|---|
| ENSG00000229356 | *LRRC3-AS1* | 1.42 | 0.033 |
| ENSG00000276107 | *AC037198.1* | 1.06 | 0.045 |
| ENSG00000270614 | *AC011451.2* | 1.35 | 0.034 |
| ENSG00000270497 | *BX322635.1* | 1.60 | 0.035 |
| ENSG00000254855 | *AP001107.8* | 1.25 | 0.032 |
| ENSG00000242085 | *RPS20P33* | 1.49 | 0.034 |
| ENSG00000259205 | *PRKXP1* | 1.47 | 0.046 |
| ENSG00000259884 | *AC025259.3* | 1.75 | 0.037 |
| ENSG00000274051 | *RF02271* | 1.14 | 0.035 |
| ENSG00000212541 | *RNU6-510P* | 1.46 | 0.038 |
| ENSG00000227666 | *CYCSP24* | 1.31 | 0.040 |
| ENSG00000238405 | *RNA5SP311* | 1.42 | 0.045 |
| ENSG00000233231 | *HNRNPA1P49* | 1.62 | 0.041 |
| ENSG00000279203 | *AC005785.2* | 1.42 | 0.046 |
| ENSG00000222112 | *RN7SKP16* | 1.18 | 0.048 |
| ENSG00000200170 | *RF00019* | 1.32 | 0.043 |
| ENSG00000202290 | *RNA5SP37* | 1.19 | 0.049 |
| ENSG00000203588 | *IGBP1-AS1* | 1.23 | 0.046 |
| ENSG00000232287 | *SLC6A1-AS1* | 1.45 | 0.044 |
| ENSG00000202533 | *RF00019* | 1.32 | 0.049 |
| ENSG00000268756 | *AC104534.1* | 1.12 | 0.046 |
| **Downregulated genes** | | | |
| ENSG00000166292 | *TMEM100* | -1.36 | 0.001 |
| ENSG00000117069 | *ST6GALNAC5* | -1.69 | 0.000 |
| ENSG00000184905 | *TCEAL2* | -1.76 | 0.002 |
| ENSG00000135744 | *AGT* | -1.38 | 0.010 |
| ENSG00000163666 | *HESX1* | -1.20 | 0.001 |
| ENSG00000158825 | *CDA* | -1.29 | 0.006 |
| ENSG00000170961 | *HAS2* | -1.23 | 0.011 |
| ENSG00000205791 | *LOH12CR2* | -1.11 | 0.002 |
| ENSG00000104435 | *STMN2* | -2.99 | 0.012 |
| ENSG00000182853 | *VMO1* | -1.70 | 0.010 |
| ENSG00000187151 | *ANGPTL5* | -1.19 | 0.011 |
| ENSG00000071991 | *CDH19* | -1.62 | 0.009 |
| ENSG00000112175 | *BMP5* | -1.90 | 0.021 |
| ENSG00000070193 | *FGF10* | -2.40 | 0.003 |
| ENSG00000172260 | *NEGR1* | -1.24 | 0.020 |
| ENSG00000066185 | *ZMYND12* | -1.08 | 0.008 |
| ENSG00000228305 | *AC016734.1* | -1.04 | 0.014 |
| ENSG00000134259 | *NGF* | -1.10 | 0.019 |
| ENSG00000258545 | *RHOXF1-AS1* | -1.28 | 0.020 |
| ENSG00000166426 | *CRABP1* | -2.79 | 0.018 |
| ENSG00000268584 | *AC073389.1* | -1.38 | 0.003 |
| ENSG00000117594 | *HSD11B1* | -2.59 | 0.016 |
| ENSG00000273368 | *AC006566.1* | -1.88 | 0.003 |
| ENSG00000138356 | *AOX1* | -1.26 | 0.028 |
| ENSG00000212664 | *AC064799.1* | -1.18 | 0.007 |

| | | | |
|---|---|---|---|
| ENSG00000125618 | *PAX8* | -1.07 | 0.030 |
| ENSG00000224043 | *CCNT2-AS1* | -1.14 | 0.009 |
| ENSG00000164669 | *INTS4P1* | -1.80 | 0.015 |
| ENSG00000284648 | *AC097493.4* | -1.49 | 0.004 |
| ENSG00000236533 | *AC009413.1* | -1.51 | 0.004 |
| ENSG00000171951 | *SCG2* | -1.89 | 0.015 |
| ENSG00000230629 | *RPS23P8* | -1.06 | 0.014 |
| ENSG00000198384 | *TPTE2P3* | -1.08 | 0.010 |
| ENSG00000231156 | *AC093702.1* | -1.10 | 0.017 |
| ENSG00000270681 | *AC095055.1* | -1.17 | 0.014 |
| ENSG00000256001 | *AC079949.1* | -1.94 | 0.009 |
| ENSG00000064886 | *CHI3L2* | -1.15 | 0.047 |
| ENSG00000272009 | *AL121944.1* | -1.38 | 0.013 |
| ENSG00000261105 | *LMO7-AS1* | -1.52 | 0.026 |
| ENSG00000253426 | *AC022784.3* | -1.06 | 0.016 |
| ENSG00000123560 | *PLP1* | -1.37 | 0.038 |
| ENSG00000168672 | *FAM84B* | -1.03 | 0.046 |
| ENSG00000278611 | *ZNF426-DT* | -1.09 | 0.024 |
| ENSG00000182327 | *GLTPD2* | -1.07 | 0.019 |
| ENSG00000243389 | *AC012442.2* | -1.39 | 0.010 |
| ENSG00000146013 | *GFRA3* | -1.99 | 0.023 |
| ENSG00000231369 | *Z97353.1* | -1.00 | 0.014 |
| ENSG00000274248 | *AJ011932.1* | -1.59 | 0.016 |
| ENSG00000250048 | *LINC00603* | -2.71 | 0.021 |
| ENSG00000272707 | *AC046143.2* | -1.37 | 0.013 |
| ENSG00000277998 | *AC107075.1* | -1.37 | 0.014 |
| ENSG00000197238 | *HIST1H4J* | -1.32 | 0.013 |
| ENSG00000255362 | *AP000785.2* | -1.41 | 0.013 |
| ENSG00000243680 | *RPL37P23* | -1.08 | 0.027 |
| ENSG00000253570 | *RNF5P1* | -1.27 | 0.016 |
| ENSG00000267114 | *AC011481.1* | -1.13 | 0.027 |
| ENSG00000268751 | *SCGB1B2P* | -1.15 | 0.036 |
| ENSG00000227077 | *AC107983.1* | -1.24 | 0.025 |
| ENSG00000271943 | *AC098614.4* | -1.22 | 0.020 |
| ENSG00000260625 | *AC026471.2* | -1.27 | 0.027 |
| ENSG00000230730 | *AC074011.1* | -1.13 | 0.026 |
| ENSG00000280502 | *RF00017* | -1.10 | 0.022 |
| ENSG00000274443 | *C8orf89* | -1.01 | 0.033 |
| ENSG00000229628 | *AC073115.1* | -1.44 | 0.027 |
| ENSG00000267079 | *AP001269.2* | -1.40 | 0.030 |
| ENSG00000070031 | *SCT* | -1.30 | 0.034 |
| ENSG00000253853 | *AC246817.1* | -1.21 | 0.032 |
| ENSG00000237797 | *AL161935.3* | -1.05 | 0.042 |
| ENSG00000162840 | *MT2P1* | -1.43 | 0.039 |
| ENSG00000200033 | *RNU6-403P* | -1.21 | 0.049 |

**Supplementary Table S4.** Significantly enriched GO terms of the DEGs in subchondral bone from patients with KBD versus OA.

| GO Term | ID | *P*-Value | Corrected *P*-value |
|---|---|---|---|
| **Biological process** | GO:0042445 | 2.51E-06 | 0.0065 |
| hormone metabolic process | GO:0042445 | 2.51E-06 | 0.0065 |
| otic vesicle development | GO:0071599 | 5E-06 | 0.0065 |
| extrinsic apoptotic signaling pathway | GO:0097191 | 7.76E-06 | 0.0067 |
| regulation of extrinsic apoptotic signaling pathway | GO:2001236 | 1.98E-05 | 0.0128 |
| regulation of hormone levels | GO:0010817 | 5.81E-05 | 0.0228 |
| negative regulation of extrinsic apoptotic signaling pathway | GO:2001237 | 6.09E-05 | 0.0228 |
| blood vessel remodeling | GO:0001974 | 9.17E-05 | 0.0228 |
| positive regulation of keratinocyte migration | GO:0051549 | 9.34E-05 | 0.0228 |
| regulation of keratinocyte migration | GO:0051547 | 9.34E-05 | 0.0228 |
| single-organism metabolic process | GO:0044710 | 9.93E-05 | 0.0228 |
| epithelial cell migration | GO:0010631 | 9.95E-05 | 0.0228 |
| epithelium migration | GO:0090132 | 0.0001 | 0.0228 |
| tissue migration | GO:0090130 | 0.0001 | 0.0239 |
| positive regulation of multicellular organismal process | GO:0051240 | 0.0001 | 0.0239 |
| positive regulation of keratinocyte proliferation | GO:0010838 | 0.0002 | 0.0258 |
| otic vesicle formation | GO:0030916 | 0.0002 | 0.0258 |
| regulation of morphogenesis of a branching structure | GO:0060688 | 0.0002 | 0.0301 |
| otic vesicle morphogenesis | GO:0071600 | 0.0002 | 0.0313 |
| cellular response to growth factor stimulus | GO:0071363 | 0.0002 | 0.0313 |
| positive regulation of cell differentiation | GO:0045597 | 0.0003 | 0.0313 |
| **Cellular component** | | | |
| extracellular region | GO:0005576 | 0.0007 | 0.0450 |
| **Molecular function** | | | |
| growth factor activity | GO:0008083 | 0.0004 | 0.0313 |
| chemoattractant activity | GO:0042056 | 0.0015 | 0.0468 |
| hydrolase activity, acting on carbon-nitrogen (but not peptide) bonds, in cyclic amidines | GO:0016814 | 0.0021 | 0.0468 |
| ATP-dependent DNA/RNA helicase activity | GO:0033680 | 0.0021 | 0.0468 |
| 5'-3' DNA/RNA helicase activity | GO:0033678 | 0.0021 | 0.0468 |
| ATP-dependent 5'-3' DNA/RNA helicase activity | GO:0033682 | 0.0021 | 0.0468 |
| type 2 angiotensin receptor binding | GO:0031703 | 0.0021 | 0.0468 |
| 11-beta-hydroxysteroid dehydrogenase (NADP+) activity | GO:0070524 | 0.0021 | 0.0468 |
| deaminase activity | GO:0019239 | 0.0023 | 0.0468 |

**Supplementary Table S5.** The downregulated miRNAs in subchondral bone from patients with KBD versus OA.

| Gene ID | Transcript ID (Array Design) | Fold Change | q-value |
|---|---|---|---|
| 20502446 | hsa-miR-451a | 0.1283 | 0.006 |
| 20501160 | hsa-miR-29c-3p | 0.1447 | 0.005 |
| 20500385 | hsa-miR-192-5p | 0.1788 | 0.006 |
| 20501206 | hsa-miR-363-3p | 0.1803 | 0.005 |
| 20504379 | hsa-miR-629-5p | 0.1859 | <0.001 |
| 20500131 | hsa-miR-17-3p | 0.1872 | <0.001 |
| 20500450 | hsa-miR-182-5p | 0.1981 | 0.005 |
| 20503105 | hsa-miR-486-5p | 0.1983 | <0.001 |
| 20500743 | hsa-miR-138-5p | 0.2122 | 0.005 |
| 20500796 | hsa-miR-193a-3p | 0.2139 | 0.005 |
| 20501300 | hsa-miR-335-5p | 0.2243 | 0.005 |
| 20500189 | hsa-miR-29b-2-5p | 0.2324 | 0.005 |
| 20529566 | hsa-miR-7975 | 0.237 | 0.005 |
| 20500188 | hsa-miR-29b-3p | 0.2494 | 0.005 |
| 20517686 | hsa-miR-4306 | 0.2536 | 0.005 |
| 20500156 | hsa-miR-27a-5p | 0.2542 | 0.005 |
| 20502235 | hsa-miR-18b-5p | 0.2569 | 0.005 |
| 20500797 | hsa-miR-194-5p | 0.2663 | 0.007 |
| 20500751 | hsa-miR-143-5p | 0.2736 | 0.005 |
| 20503106 | hsa-miR-486-3p | 0.2738 | 0.005 |
| 20515591 | hsa-miR-1260b | 0.281 | 0.005 |
| 20501291 | hsa-miR-148b-3p | 0.2838 | 0.005 |
| 20501159 | hsa-miR-29c-5p | 0.2846 | 0.005 |
| 20500154 | hsa-miR-26b-5p | 0.2869 | 0.005 |
| 20502451 | hsa-miR-452-5p | 0.2884 | 0.005 |
| 20518919 | hsa-miR-4521 | 0.2889 | 0.005 |
| 20504431 | hsa-miR-660-5p | 0.2913 | 0.005 |
| 20517744 | hsa-miR-4284 | 0.2981 | 0.005 |
| 20517704 | hsa-miR-4324 | 0.3026 | 0.005 |
| 20500465 | hsa-miR-210-3p | 0.3122 | 0.005 |
| 20500132 | hsa-miR-18a-5p | 0.3149 | 0.005 |
| 20502237 | hsa-miR-20b-5p | 0.3177 | 0.005 |
| 20500724 | hsa-miR-30b-5p | 0.3288 | 0.005 |
| 20500488 | hsa-miR-223-3p | 0.3337 | 0.005 |
| 20501772 | hsa-miR-196b-3p | 0.3337 | 0.006 |
| 20517745 | hsa-miR-4286 | 0.3342 | <0.001 |
| 20501182 | hsa-miR-30e-5p | 0.3405 | 0.005 |

| | | | |
|---|---|---|---|
| 20500438 | hsa-miR-10a-5p | 0.3414 | <0.001 |
| 20500126 | hsa-miR-15a-5p | 0.3416 | 0.005 |
| 20518834 | hsa-miR-4454 | 0.3421 | 0.005 |
| 20504580 | hsa-miR-769-5p | 0.3481 | 0.005 |
| 20521811 | hsa-miR-664b-3p | 0.3484 | 0.046 |
| 20503809 | hsa-miR-497-5p | 0.3504 | <0.001 |
| 20528493 | hsa-miR-7641 | 0.3515 | 0.010 |
| 20504584 | hsa-miR-378d | 0.3552 | <0.001 |
| 20500752 | hsa-miR-143-3p | 0.3567 | 0.005 |
| 20500443 | hsa-miR-34a-3p | 0.3582 | 0.006 |
| 20518783 | hsa-miR-378e | 0.3584 | 0.005 |
| 20500161 | hsa-miR-29a-3p | 0.3646 | 0.005 |
| 20504407 | hsa-miR-652-5p | 0.3677 | 0.005 |
| 20518794 | hsa-miR-378g | 0.3686 | 0.005 |
| 20500490 | hsa-miR-224-3p | 0.3687 | 0.007 |
| 20500423 | hsa-miR-30c-2-3p | 0.3701 | <0.001 |
| 20504325 | hsa-miR-550a-3p | 0.3705 | 0.005 |
| 20500149 | hsa-miR-24-2-5p | 0.3733 | 0.005 |
| 20518936 | hsa-miR-378i | 0.3734 | 0.005 |
| 20500787 | hsa-miR-185-5p | 0.3736 | 0.005 |
| 20501183 | hsa-miR-30e-3p | 0.3756 | 0.005 |
| 20500157 | hsa-miR-27a-3p | 0.382 | 0.005 |
| 20501299 | hsa-miR-339-3p | 0.3841 | 0.020 |
| 20518788 | hsa-miR-378f | 0.3876 | 0.006 |
| 20500478 | hsa-miR-218-5p | 0.389 | 0.009 |
| 20501294 | hsa-miR-324-5p | 0.3897 | <0.001 |
| 20500194 | hsa-miR-106a-5p | 0.3908 | <0.001 |
| 20501278 | hsa-miR-328-3p | 0.3912 | <0.001 |
| 20500432 | hsa-miR-139-5p | 0.3919 | 0.005 |
| 20501292 | hsa-miR-331-5p | 0.3942 | 0.005 |
| 20502122 | hsa-miR-422a | 0.3944 | <0.001 |
| 20500452 | hsa-miR-183-5p | 0.3952 | 0.005 |
| 20500433 | hsa-miR-139-3p | 0.3961 | 0.005 |
| 20500130 | hsa-miR-17-5p | 0.3979 | 0.005 |
| 20500137 | hsa-miR-19b-3p | 0.4 | 0.005 |
| 20501157 | hsa-miR-106b-5p | 0.4028 | <0.001 |
| 20500163 | hsa-miR-30a-3p | 0.4037 | 0.005 |
| 20504408 | hsa-miR-652-3p | 0.4086 | 0.006 |
| 20500739 | hsa-miR-133a-3p | 0.4095 | 0.005 |
| 20500162 | hsa-miR-30a-5p | 0.4098 | 0.005 |
| 20500483 | hsa-miR-221-5p | 0.4115 | 0.005 |
| 20500489 | hsa-miR-224-5p | 0.4116 | 0.005 |

| | | | |
|---|---|---|---|
| 20517814 | hsa-miR-3607-5p | 0.4132 | 0.005 |
| 20503794 | hsa-miR-146b-3p | 0.4146 | <0.001 |
| 20517675 | hsa-miR-378c | 0.4154 | 0.005 |
| 20500143 | hsa-miR-22-5p | 0.4158 | <0.001 |
| 20501201 | hsa-miR-362-5p | 0.4175 | 0.006 |
| 20503888 | hsa-miR-513a-5p | 0.42 | 0.005 |
| 20500182 | hsa-miR-99a-3p | 0.4261 | <0.001 |
| 20503793 | hsa-miR-146b-5p | 0.431 | 0.005 |
| 20503786 | hsa-miR-489-3p | 0.4356 | <0.001 |
| 20529568 | hsa-miR-7977 | 0.4356 | 0.008 |
| 20500181 | hsa-miR-99a-5p | 0.4398 | 0.005 |
| 20500755 | hsa-miR-145-5p | 0.4412 | <0.001 |
| 20500179 | hsa-miR-98-5p | 0.4416 | 0.006 |
| 20501242 | hsa-miR-378a-5p | 0.4417 | 0.006 |
| 20502129 | hsa-miR-425-5p | 0.4433 | 0.006 |
| 20500445 | hsa-miR-181a-2-3p | 0.4433 | 0.005 |
| 20500133 | hsa-miR-18a-3p | 0.4473 | <0.001 |
| 20500139 | hsa-miR-20a-5p | 0.4497 | 0.005 |
| 20500782 | hsa-miR-150-5p | 0.4511 | <0.001 |
| 20518855 | hsa-miR-4470 | 0.4519 | <0.001 |
| 20500778 | hsa-miR-146a-5p | 0.4598 | 0.005 |
| 20501243 | hsa-miR-378a-3p | 0.4615 | 0.005 |
| 20501771 | hsa-miR-196b-5p | 0.4638 | 0.005 |
| 20506815 | hsa-miR-1287-5p | 0.4639 | <0.001 |
| 20507089 | hsa-miR-513b-5p | 0.4645 | 0.005 |
| 20503789 | hsa-miR-491-5p | 0.4674 | 0.005 |
| 20500421 | hsa-miR-148a-3p | 0.4713 | <0.001 |
| 20500424 | hsa-miR-30d-5p | 0.4714 | 0.005 |
| 20500144 | hsa-miR-22-3p | 0.4729 | 0.005 |
| 20511560 | hsa-miR-2116-5p | 0.4747 | <0.001 |
| 20500183 | hsa-miR-100-5p | 0.4756 | <0.001 |
| 20500735 | hsa-miR-130a-3p | 0.4765 | 0.005 |
| 20500142 | hsa-miR-21-3p | 0.477 | <0.001 |
| 20500173 | hsa-miR-93-5p | 0.4811 | <0.001 |
| 20505760 | hsa-miR-708-5p | 0.4826 | <0.001 |
| 20500725 | hsa-miR-30b-3p | 0.4851 | <0.001 |
| 20500731 | hsa-miR-125b-1-3p | 0.4866 | <0.001 |
| 20501312 | hsa-miR-345-5p | 0.4882 | <0.001 |
| 20500798 | hsa-miR-195-5p | 0.4897 | <0.001 |
| 20500174 | hsa-miR-93-3p | 0.4916 | 0.007 |
| 20501036 | hsa-miR-200c-3p | 0.4949 | 0.007 |

| | | | |
|---|---|---|---|
| 20500196 | hsa-miR-107 | 0.4951 | 0.007 |
| 20500756 | hsa-miR-145-3p | 0.4956 | <0.001 |
| 20503102 | hsa-miR-484 | 0.4994 | <0.001 |
| 20500723 | hsa-miR-27b-3p | 0.4995 | <0.001 |

**Supplementary Table S7.** miRNA-target gene interaction pairs of the downregulated miRNA and upregulated targeted genes.

| miRNA | accession | Gene Symbol |
|---|---|---|
| hsa-miR-146b-3p | MIMAT0004766 | *ADAMTS14* |
| hsa-miR-18a-3p | MIMAT0002891 | *ADAMTS14* |
| hsa-miR-193a-3p | MIMAT0000459 | *ADAMTS14* |
| hsa-miR-218-5p | MIMAT0000275 | *ADAMTS14* |
| hsa-miR-221-5p | MIMAT0004568 | *ADAMTS14* |
| hsa-miR-27a-3p | MIMAT0000084 | *ADAMTS14* |
| hsa-miR-27b-3p | MIMAT0000419 | *ADAMTS14* |
| hsa-miR-29a-3p | MIMAT0000086 | *ADAMTS14* |
| hsa-miR-30c-2-3p | MIMAT0004550 | *ADAMTS14* |
| hsa-miR-324-5p | MIMAT0000761 | *ADAMTS14* |
| hsa-miR-328-3p | MIMAT0000752 | *ADAMTS14* |
| hsa-miR-339-3p | MIMAT0004702 | *ADAMTS14* |
| hsa-miR-4324 | MIMAT0016876 | *ADAMTS14* |
| hsa-miR-486-3p | MIMAT0004762 | *ADAMTS14* |
| hsa-miR-489-3p | MIMAT0002805 | *ADAMTS14* |
| hsa-miR-513a-5p | MIMAT0002877 | *ADAMTS14* |
| hsa-miR-98-5p | MIMAT0000096 | *ADAMTS14* |
| hsa-miR-145-3p | MIMAT0004601 | *CEACAM1* |
| hsa-miR-146a-5p | MIMAT0000449 | *CEACAM1* |
| hsa-miR-146b-5p | MIMAT0002809 | *CEACAM1* |
| hsa-miR-148a-3p | MIMAT0000243 | *CEACAM1* |
| hsa-miR-148b-3p | MIMAT0000759 | *CEACAM1* |
| hsa-miR-17-3p | MIMAT0000071 | *CEACAM1* |
| hsa-miR-182-5p | MIMAT0000259 | *CEACAM1* |
| hsa-miR-183-5p | MIMAT0000261 | *CEACAM1* |
| hsa-miR-18a-5p | MIMAT0000072 | *CEACAM1* |
| hsa-miR-18b-5p | MIMAT0001412 | *CEACAM1* |
| hsa-miR-19b-3p | MIMAT0000074 | *CEACAM1* |
| hsa-miR-218-5p | MIMAT0000275 | *CEACAM1* |
| hsa-miR-221-5p | MIMAT0004568 | *CEACAM1* |
| hsa-miR-224-3p | MIMAT0009198 | *CEACAM1* |
| hsa-miR-29a-3p | MIMAT0000086 | *CEACAM1* |
| hsa-miR-29b-2-5p | MIMAT0004515 | *CEACAM1* |
| hsa-miR-29b-3p | MIMAT0000100 | *CEACAM1* |
| hsa-miR-29c-3p | MIMAT0000681 | *CEACAM1* |
| hsa-miR-30a-5p | MIMAT0000087 | *CEACAM1* |
| hsa-miR-30b-5p | MIMAT0000420 | *CEACAM1* |
| hsa-miR-30d-5p | MIMAT0000245 | *CEACAM1* |
| hsa-miR-30e-5p | MIMAT0000692 | *CEACAM1* |
| hsa-miR-378a-5p | MIMAT0000731 | *CEACAM1* |
| hsa-miR-378g | MIMAT0018937 | *CEACAM1* |
| hsa-miR-4470 | MIMAT0018997 | *CEACAM1* |

| | | |
|---|---|---|
| hsa-miR-486-3p | MIMAT0004762 | *CEACAM1* |
| hsa-miR-629-5p | MIMAT0004810 | *CEACAM1* |
| hsa-miR-15a-5p | MIMAT0000068 | *HSPA1B* |
| hsa-miR-195-5p | MIMAT0000461 | *HSPA1B* |
| hsa-miR-497-5p | MIMAT0002820 | *HSPA1B* |
| hsa-miR-497-5p | MIMAT0002820 | *HSPA1B* |
| hsa-miR-145-5p | MIMAT0000437 | *PIF1* |
| hsa-miR-497-5p | MIMAT0002820 | *PIF1* |
| hsa-miR-629-5p | MIMAT0004810 | *PIF1* |
| hsa-miR-185-5p | MIMAT0000455 | *SIGLEC14* |
| hsa-miR-27a-3p | MIMAT0000084 | *SIGLEC14* |
| hsa-miR-27b-3p | MIMAT0000419 | *SIGLEC14* |
| hsa-miR-486-3p | MIMAT0004762 | *SIGLEC14* |
| hsa-miR-513a-5p | MIMAT0002877 | *SIGLEC14* |
| hsa-miR-513b-5p | MIMAT0005788 | *SIGLEC14* |
| hsa-miR-769-5p | MIMAT0003886 | *SIGLEC14* |
| hsa-miR-98-5p | MIMAT0000096 | *SIGLEC14* |
| hsa-miR-143-3p | MIMAT0000435 | *SLC13A5* |
| hsa-miR-145-3p | MIMAT0004601 | *SLC13A5* |
| hsa-miR-146b-3p | MIMAT0004766 | *SLC13A5* |
| hsa-miR-150-5p | MIMAT0000451 | *SLC13A5* |
| hsa-miR-15a-5p | MIMAT0000068 | *SLC13A5* |
| hsa-miR-182-5p | MIMAT0000259 | *SLC13A5* |
| hsa-miR-185-5p | MIMAT0000455 | *SLC13A5* |
| hsa-miR-18a-3p | MIMAT0002891 | *SLC13A5* |
| hsa-miR-195-5p | MIMAT0000461 | *SLC13A5* |
| hsa-miR-22-3p | MIMAT0000077 | *SLC13A5* |
| hsa-miR-26b-5p | MIMAT0000083 | *SLC13A5* |
| hsa-miR-30b-3p | MIMAT0004589 | *SLC13A5* |
| hsa-miR-30c-2-3p | MIMAT0004550 | *SLC13A5* |
| hsa-miR-335-5p | MIMAT0000765 | *SLC13A5* |
| hsa-miR-497-5p | MIMAT0002820 | *SLC13A5* |
| hsa-miR-629-5p | MIMAT0004810 | *SLC13A5* |
| hsa-miR-708-5p | MIMAT0004926 | *SLC13A5* |
